# MAP kinase kinase 1 (MEK1) within extracellular vesicles inhibits tumour growth by promoting anti‐tumour immunity

**DOI:** 10.1002/jev2.12515

**Published:** 2024-09-27

**Authors:** Stephen C. Searles, Wei‐Shan Chen, Jarrod D. Yee, Preston Lee, Calvin K. Lee, Christine Caron, Felippe Neto, Irina Matei, David Lyden, Jack D. Bui

**Affiliations:** ^1^ Department of Pathology University of California San Diego California USA; ^2^ Department of Cell and Developmental Biology Weill Cornell Medical College New York New York USA

**Keywords:** extracellular vesicles, macrophages, MEK1, regressors

## Abstract

Extracellular vesicles (EVs) mediate intercellular communication in many physiologic processes and can modulate immune responses in individuals with cancer. Most studies of EVs in cancer have focused on their tumour promoting properties. Whether and how EVs might mediate tumour regression besides carrying antigens has not been well characterized. Using a mouse model of highly immunogenic regressor versus poorly immunogenic progressor tumour cells, we have characterized the role of EVs in activating macrophages and promoting tumour rejection. We found that the signalling molecule MAP2K1 (MEK1) is enriched in EVs secreted by regressor relative to progressor cells. Progressor EVs engineered to have levels of MEK1 similar to regressor EVs could inhibit tumour growth by indirectly promoting adaptive immunity in both syngeneic and 3rd party tumours. This effect required MEK1 activity and could occur by activating macrophages to promote adaptive immune responses against the tumour via the cytokine interferon‐gamma. Our results suggest that MEK inhibition may be deleterious to cancer treatment, since MEK1 plays an important cell‐extrinsic, tumour‐suppressive role within EVs. Moreover, the delivery of MEK1 to tumour‐associated macrophages, either by EVs, nanoparticles, or some other means, could be a useful strategy to treat cancer via the activation of anti‐tumour immunity.

## 1 INTRODUCTION

All cells have the capability to produce and release EVs, small vesicles that have similar topology to the cell and can deliver diverse cargo to mediate cell–cell communication (Kalluri & LeBleu, 2020; Mathieu et al., 2019; Pegtel & Gould, 2019; Tkach & Thery, 2016; van Niel et al., 2018; Wortzel et al., 2019). EVs derive from the late endosomal pathway and contain bioactive cargo that can include lipids, nucleic acids (both RNA and DNA), and proteins. Once released into the extracellular space, EVs can bind to and enter other cells, resulting in the physical transfer of bioactive cargo between cells, leading to functional changes in the target cell (Peinado et al., 2012; Valadi et al., 2007; Wolfers et al., 2001).

Cancer cells secrete more EVs than non‐transformed cells of the same type and can deliver cancer antigens to immune cells (Andre et al., 2002, 2002; Koga et al., 2005; Raposo et al., 1996; Wolfers et al., 2001), leading to immune‐stimulatory effects. On the other hand, tumour derived (TD)‐EVs can also exert inhibitory effects on immune cells and promote tumour growth (Valenti et al., 2007; Zhang & Grizzle, 2011). For example, TD‐EVs laden with Fas‐ligand can induce apoptosis of tumour‐specific CD8+ T cells (Abusamra et al., 2005; Albanese et al., 1998; Kim et al., 2005). In another example, MET protein within melanoma EVs has been shown to augment metastasis by permanently educating bone marrow progenitor cells to promote metastatic niche formation (Peinado et al., 2012).

Previous studies of EVs from cancers focused on progressively growing tumours that typically induce immune suppression. We have used a model system of highly immunogenic regressor and poorly immunogenic progressor cells to elucidate the pathways of tumour rejection (O'Sullivan et al., 2012, 2014; Shankaran et al., 2001), identifying T cells, interferons (IFNs), and natural killer (NK) cells as important for rejection, but we have not examined EVs from regressor cell lines. In this report, we document the protein differences in EVs produced by regressor versus progressor cells. We have found MAP2K1 (MEK1) as a functional protein in regressor EVs that could activate tumour‐associated macrophages to initiate an interferonγ (IFNγ)‐dependent anti‐tumour immune response.

## 2 MATERIALS AND METHODS

### 2.1 Study design

Experiments in which a continuous variable outcome was expected (i.e., animal growth, flow cytometry, transcript measurements) used power calculations to guide the quantity required for each cohort to demonstrate a 1.5‐fold difference in signal with an alpha value of 0.05 and a power of 0.8. Experiments in which a dichotomous outcome was expected (i.e., proteomic analysis, RNAseq) were assessed retrospectively and judgment was used to identify unique protein/gene differences.

### 2.2 Mice

Wild‐type C57BL/6 and RAG1^−/−^ mice were purchased from Jackson Laboratories. All experiments involving mice were conducted under the animal protocol approved by the University of California, San Diego Institutional Animal Care and Use Committee (IACUC protocol #S06201). For EV therapy, 20 µg EVs were administered via intratumoural injection. To block MEK1 activity in vivo, the MEK1 inhibitor PD98059 (Cell Signalling Technology, Danvers, MA, USA) was used at 10 mg/kg (Di Paola et al., 2010). IFNγ blockade and NK cell depletion was achieved by injection of H22 or PK136 antibody, respectively (Biolegend, San Diego, CA USA), as previously described (O'Sullivan et al., 2012). Clodronate liposomes were used to deplete macrophages according to the manufacturer's recommendations (Liposoma BV, Amsterdam, Netherlands).

### 2.3 Tumour transplantation

Tumour cell lines were grown in vitro (as described below), harvested by trypsinization, washed three times, and injected subcutaneously into the flank of mice at 1–5 million cells (O'Sullivan et al., 2012). For studies analysing tumour‐infiltrating immune cells, after sacking mice, tumours were processed into a single cell suspension by mechanical dicing/collagenase digestion as previously described (O'Sullivan et al., 2012) and analysed by FACS.

### 2.4 Cell lines and culture conditions

MCA sarcoma cells and B16 melanoma cells were cultured in RPMI 1640 supplemented with 10% foetal bovine serum, 1 mM sodium pyruvate, 0.0375% sodium bicarbonate, 5% MEM non‐essential amino acids, 2 mM l‐glutamine, 10 µg/mL ciprofloxacin, and 56 µM 2‐mercaptoethanol (O'Sullivan et al., 2012). 4862, 6727, d42m1, H31m1, 9609, 9614, d29m1, and F236 MCA sarcoma cell lines were generated previously (O'Sullivan et al., 2012; Shankaran et al., 2001).

### 2.5 Viral transduction of cancer cell lines

For GFP, MEK1, and HA‐MEK1 expression in 9609 cells, retroviruses generated from 293T cells were used to transduce parental 9609 cells. Retroviral plasmids for MEK1 (pLZRS‐Mek1‐wt1 #21196) and HA‐MEK1 (pBABEpuro‐HA‐MEK1 #53195) were purchased from AddGene (Cambridge, MA, USA). The retroviral plasmid for GFP (pMSCV‐GFP) was provided by Dr. Robert Schreiber (Bui et al., 2006).

### 2.6 Isolation of EVs

EVs were isolated by differential ultracentrifugation as previously described (Thery et al., 2006). Briefly, cells were grown for 48–72 h in media containing serum that had been depleted of EVs. The conditioned media was harvested and subjected to serial centrifugation: 10 min at 500 × *g* (to remove large debris/dead cells) followed by 20 min at 20,000 × *g* (to remove small debris/apoptotic bodies). Next, the cleared conditioned media was spun for 70 min at 100,000 × *g* to pellet EVs. The EV pellet was resuspended in a large volume of HBSS and spun again for 70 min at 100,000 × *g* to wash soluble proteins from the EVs. Finally, the washed pellet was resuspended in HBSS in a volume approximately 1/500th of the starting volume of conditioned media. The concentration was determined by BCA assay (Thermo Fisher, Waltham, MA, USA). All ultracentrifugation steps were performed using a Beckman Avanti J‐30I ultracentrifuge with a JA‐30.50 Ti fixed‐angle rotor (Beckman Coulter, Carlsbad, CA, USA).

### 2.7 Proteomic analysis of EVs

**FIGURE 1:**
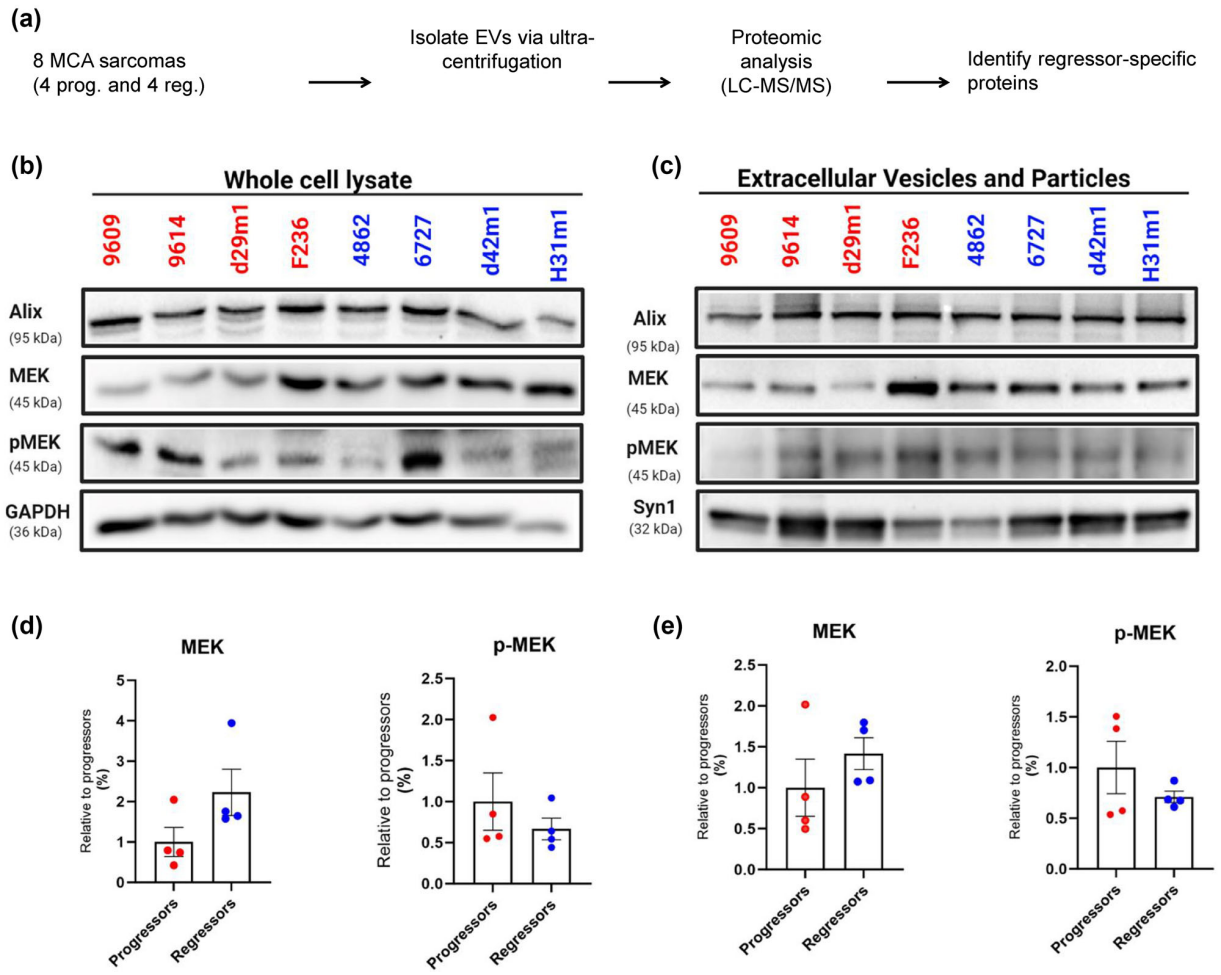
MEK1 (MP2K1) is enriched in regressor EVs. (a) Schematic representation depicting workflow for proteomic analysis of progressor and regressor EVs. (b and c) Western blot analysis of MEK1, phosphorylated MEK1 (pMEK1, Ser298), Alix, GAPDH, and Syn in (b) whole cell lysate and (c) extracellular vesicles from progressor and regressor cell lines. (d,e) Quantified and normalized expression of individual protein bands is shown in regressor and progressor groups in (D) WCL and (e) EV blots. 10 µg of protein was loaded in each lane. Also shown is quantification of MEK1 and pMEK1 abundance in EVs, normalized against Alix. Each symbol represents a single progressor or regressor cell line, and the horizontal line represents the mean of each group. Data is representative of three independent experiments. See also Figures S2–S3 and Tables S1–S2.

Proteomic analysis of EVs via mass spectrometry was performed as previously described (Hoshino et al., 2015). About 10–20 µg of EV protein was denatured, reduced, and alkylated, followed by proteolytic digestion with endoproteinase and trypsin. For analysis by reverse phase liquid chromatography‐tandem mass spectrometry (LC‐MS/MS), approximately 3–5 µg of each sample was loaded onto a C18 trap column with 5 µm beads, and peptides were separated using a 75‐µm inner diameter C18 column with 3 µm beads. Each spectra obtained was extracted and searched against the Uniprot mouse proteome database based on a 1% false discovery rate. To quantify protein amounts in each sample, the average area of the three most abundant peptides for each matched protein was used. For each analysis, data from three technical replicates was used. The proteomic study in Figures 1 and S2 were non‐quantitative, whereas the study in Figure S5 was semi‐quantitative.

### 2.8 Immunoblotting

EVs or whole cells were lysed in sample buffer (Morganville Scientific, #LB0100) containing 10% β‐mercaptoethanol (Sigma‐Aldrich, #M6250‐10 M). SDS‐PAGE was performed under reducing conditions by loading 10 µg of protein onto a 4%–20% Tris‐Glycine Gel (NovexTM WedgeWellTM Tris‐Glycine Gel, #XP04200BOX). The proteins were then transferred to a PVDF membrane, and the membrane was blocked for 2 h in 5% BSA‐TBST 0.1% solution, incubated overnight with primary antibodies diluted 1:1000 in 5% BSA TBST 0.1% (Recombinant monoclonal Alix [EPR23653‐32]—Abcam; Rabbit polyclonal Syntenin1 [ab19903]—Abcam; Recombinant monoclonal GAPDH [#2118]—Cell Signalling; Recombinant monoclonal MEK [D2R10]—Cell Signalling; Rabbit polyclonal Phospho‐MEK1/2 [#9121]—Cell Signalling), followed by 1 h incubation with HRP conjugated Rabbit anti‐IgG (1:10000 in 5% BSA TBST 0.1%). Imaging was performed by adding chemiluminescent substrate for HRP detection (PierceTM ECL Western blotting Substrate, ThermoScientific #32106; and SuperSignalTM West Femto Maximum Sensitivity Substrate,ThermoScientific, #34095) and images were obtained using ChemiDoc XRS+ (Biorad, California, USA). Densitometry analyses were performed by using ImageLab 6.1 (Biorad, California, USA). For whole cell lysate (WCL) samples, MEK and p‐MEK signal were normalized by GAPDH, and for EV samples normalization was done by Alix. Results are expressed relative to progressors. Data are presented as mean ± SEM. Unpaired *t*‐test with Welch's correction was applied in order to detect differences among the groups, and significance was assumed when *p* < 0.05.

### 2.9 EV characterization

The size distribution and production amount of EVs was analysed by nanoparticle tracking analysis. The size and morphology of EVs was evaluated by transmission electron microscopy using a previously described method (Thery et al., 2006). EVs were stained with 2% uranyl acetate for 1 min, and grids were viewed using a JEOL 1200EX II (JOEL, Peabody, MA, USA) transmission electron microscope and photographed using a Gatan digital camera (Gatan, Pleasanton, CA, USA).

### 2.10 Flow cytometry and antibodies

Antibodies used include: APC‐Cy7‐conjugated anti‐CD45 (clone #30‐F11, Biolegend, San Diego, CA, USA), PE‐conjugated anti‐CD45 (clone #104, Biolegend), PE‐Cy7‐conjugated anti‐F4/80 (clone #BM8, Biolegend), APC‐conjugated anti‐MHCII (clone #M5/114.15.2, Biolegend), PE‐Cy7‐conjugated anti‐CD11b (clone #M1/70, eBioscience, San Diego, CA, USA), APC‐conjugated anti‐Ly6C (clone #HK1.4, Biolegend), PE‐conjugated anti‐CD4 (clone #GK1.5, Biolegend), APC‐conjugated anti‐CD8 (clone #53‐6.7, Biolegend), APC‐conjugated anti‐NK1.1 (clone #PK136, Biolegend), PE‐Cy7‐conjugated anti‐CD3 (clone #17A.2, Biolegend), PE‐Cy7‐conjugated anti‐CD11c (clone #N418, eBioscience), PE‐Cy7‐conjugated anti‐Sca1 (clone #D7, Biolegend), and AlexaFluor647‐conjugated anti‐CD31 (clone #390, Biolegend). Surface staining was performed in the presence of 1 µg/mL F_c_ blocking anti‐CD16/32 antibody. 7‐aminoactinomycin D (7‐AAD, Calbiochem, San Diego, CA, USA) was added immediately before FACS analysis at a final concentration of 1 µg/mL to stain and exclude dead cells. FACS was performed using a BD FACSCanto (BD, Franklin Lakes, NJ, USA) and data were analysed using FlowJo software (Treestar, Ashland, OR, USA).

### 2.11 Fluorescent labelling of EVs

As described (Pospichalova et al., 2015), EVs were incubated with CFSE at a final concentration of 25 µM for 30 min at 37°C in the dark. After 30 min of labelling, excess CFSE was washed out by spinning the EVs in a large volume of HBSS for 70 min at 100,000 × *g*. EVs were resuspended in a volume approximately 1/500th the original starting volume of conditioned media.

### 2.12 Quantitative RT‐PCR

RNA was extracted from cell lines using TRIzol reagent (Life Technologies, Carlsbad, CA, USA) and measured with a ND100 spectrophotometer (Nanodrop, Wilmington, DE, USA) for concentration and purity. RNA was then subjected to cDNA synthesis using High Capacity cDNA Reverse Transcription Kit (Applied Biosystem, Foster City, CA, USA) according to the manufacturer's protocol. qPCR was performed using SYBR Green PCR Master Mix (Applied Biosystem, Foster City, CA, USA) and the following thermal cycle conditions: 10 min at 95°C, followed by forty cycles consisting of: 10 s at 95°C, 60 s at 60°C using a CFX96 Touch Real‐Time PCR Detection System (Bio‐Rad Laboratories, Irvine, CA, USA). Gene expression was analysed with the 2^−ΔΔCt^ method normalized against *Hprt*. Primer sequences are listed in Table S.

### 2.13 RNA sequencing volcano plot and GSEA analysis

For RNA sequencing, paired‐end sequence files were generated from the Illumina NovaSeq 6000 (UC San Diego IGM Genomics Center). All subsequent quality control, read alignment, transcript assembly, and differential gene expression analyses were performed on Galaxy, an open source web‐based platform for bioinformatic analyses. After uploading paired‐end sequence files to Galaxy, all files were initially quality controlled using FastQC (Galaxy Version 0.73+galaxy0). Quality controlled reads were aligned with TopHat2 (Version 2.1.1) using mm10 reference genome to generate accepted_hits. Next, transcript assemblies were created from accepted_hits using Cufflinks (Galaxy Version 2.2.1.3) with reference annotation gtf file input from Gencode Release M25 (GRCm38.p6). Transcript assemblies from all samples in a dataset were then merged into one file using Cuffmerge (Galaxy Version 0.0.6). Finally, the merged file was input to Cuffdiff (Galaxy Version 2.2.1.6). The Cuffdiff gene differential expression testing output file was downloaded from Galaxy and viewed on Microsoft Excel. Fold change was calculated in MEK1‐hi relative to MEK1‐low experimental treatment. Volcano plots displaying log_2_(fold_change) and ‐log(FDR) were created using GraphPad Prism 8, with genes of interest indicated at FDR < 0.05. For running GSEA (version 4.2.3) analyses, expression dataset and phenotype label files were created from gene differential expression output files generated from Cuffdiff containing gene IDs and FPKM gene counts. The following specified parameters were used: Gene sets database, Hallmarks; Permutation type, phenotype; Chip platform, Mouse_ENSEMBL_Gene_ID_Human_Orthologs_MSigDB.v7.4.chip; Metric for ranking genes, log2_Ratio_of_Classes. For reading GSEA results, gsea_report files were generated and enriched gene sets of interest were determined by Normalized Enrichment Score (NES) > 1 and FDR q‐val < 0.01.

### 2.14 Quantification and statistical analysis

GraphPad Prism 7 (GraphPad Software, La Jolla, CA, USA) was used to analyse all datasets. Pairwise comparisons were generated with two‐tailed *t* tests. Variance between groups was calculated with the F test using a confidence level of *α* = 0.01. Definitions of centre/dispersion measurements and *n* values are all indicated in the associated figure legends for each figure. *P*‐values are represented as follows: *p < 0.05, **p < 0.01, ***p < 0.001, ****p < 0.0001.

## 3 RESULTS

### 3.1 MEK1 is enriched in regressor EVs

We previously generated and characterized a set of immunogenic 3′methylcholanthrene (MCA)‐induced “regressor” (O'Sullivan et al., 2012; Shankaran et al., 2001) and matched MCA‐induced “progressor” cell lines derived under similar conditions. Regressor cell lines cannot grow well when transplanted into syngeneic wild‐type (WT) mice but can grow when transplanted into immune deficient RAG1^−/−^ mice (O'Sullivan et al., 2012; Shankaran et al., 2001) (Figure S1), whereas progressor cell lines model typical tumours that grow progressively in immune competent hosts. Regressor and progressor cell lines are generated from individual mice and do not share antigens and cannot cross‐immunize (O'Sullivan et al., 2012, 2014; Shankaran et al., 2001). We hypothesized that EVs from regressor cell lines could mediate immune activation and provide a mechanism by which regressors undergo immune rejection, independent of carrying regressor antigens. Therefore, we characterized EVs harvested from in vitro cultured independent regressor (*n* = 4) and progressor (*n* = 4) cell lines using proteomic analysis (Figure 1a) (Thery et al., 2006). We identified proteins common to progressors (205 proteins), regressors (254 proteins), and both (155 proteins) (Figure S2 and Table S1). Comparing our protein list with publicly available data, we found that of the 25 most commonly identified proteins in the ExoCarta database ((http://exocarta.org/), 72% (Kim et al., 2005) belonged to the fibrosarcoma EV signature and the rest were detected in most of the EV preparations (Figure S3), validating our proteomic approach.

We next performed a comparative analysis to identify proteins that were present in regressor EVs and absent in progressor EVs. We established a list of nine differentially expressed candidate proteins (Table S2). Interestingly, only one protein, MAP2K1 (MEK1), was predicted to be present in four out of four regressor EVs and absent in four out of four progressor EVs.

We next attempted to verify our proteomic findings via western blot, examining MEK1 and phosphorylated MEK1 (pMEK1) in the whole cell lysate (WCL) and EVs of regressor and progressor cell lines. We found that MEK1 tended to be higher on average in WCLs and EVs from regressor compared to progressor cells (Figure 1b–e). This did not correlate with pMEK1 levels, which showed no significant differences in the WCL or EVs from regressor versus progressor cell lines. Overall, the western blot data show that three of the four progressor cell lines had levels of MEK1 lower than the average regressor cell line, implicating this gene in mediating potential systemic effects of EVs on immunity. One progressor cell line, F236, had levels of MEK1 in the WCL and EVs similar to regressors, suggesting that MEK1 expression level is not sufficient to serve as a biomarker of whether a tumour is a regressor or progressor and that other characteristics such as mutational burden or production of immune suppressive molecules need to be taken into account.

### 3.2 Overexpression of MEK1 in progressor tumours increases the MEK1 content in progressor EVs without appreciably changing content, size, or secretion level of EVs

**FIGURE 2:**
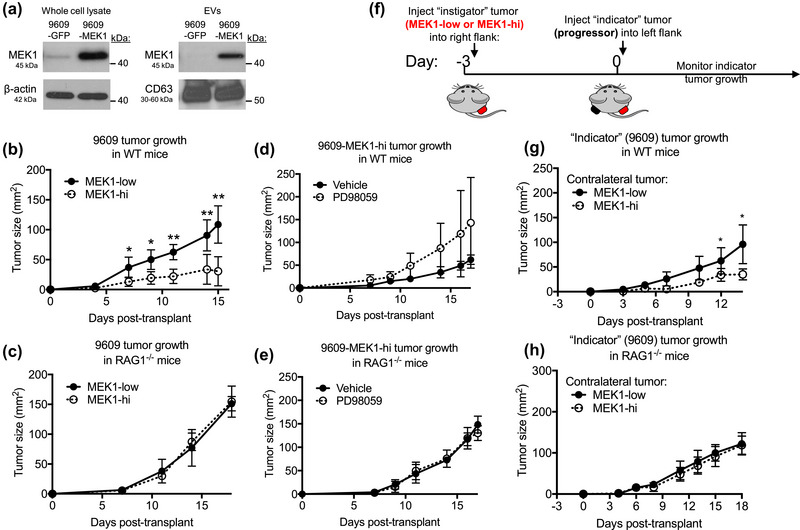
Forced expression of MEK1 in progressor tumours delays tumour growth via secreted factors and adaptive immunity, requiring MEK1 activity. (a) Western blot analysis of whole cell lysate and EVs from the progressor cell line 9609 transduced with GFP‐ or MEK1‐expressing retroviruses. MEK1 overexpression extended to the EV compartment. CD63 was used to validate the EV fraction. About 10 µg of protein was loaded in each lane. (b, c) Growth curves of 9609‐GPF (MEK1‐low) or 9609‐MEK1 (MEK1‐hi) tumours grown in (b) WT or (c) RAG1^‐/‐^ mice. Growth curves of (b) 9609‐MEK1‐low and (c) 9609‐MEK1‐hi tumours grown in WT mice and treated with PD98059 or vehicle control as described above. (d and e) Growth curves of (d) 9609‐MEK1‐hi grown in (d) WT or (e) RAG1^‐/‐^ mice and treated with PD98059 or vehicle control. (f) Schematic representation of workflow for contralateral flank experiment. “Instigator” tumour cells (1e6 9609‐GFP or 9609‐MEK1 cells) were injected s.c. into the right flank of mice at day −3. At day 0, “indicator” tumour cells (1e6 9609 cells) were injected s.c. into the left flank, and tumour growth was monitored over time. Growth curves of indicator progressor tumours (9609) grown contralaterally with instigator tumours (9609‐GFP or 9609‐MEK1) in (g) WT and (h) RAG1^‐/‐^ mice. *n* = 4–6 animals per group. Data are represented as mean ± SD. See also Figures S4–S7.

We were intrigued that MEK1, a proto‐oncogene, showed this expression pattern, since we had anticipated finding tumour‐suppressive proteins in regressor EVs. Therefore, we decided to further investigate the function of this regressor candidate protein in EVs. We accomplished this by overexpressing MEK1 in the progressor cell line 9609 (which has very low MEK1 expression in EVs, Figure 1b) via retroviral transduction. 9609 cells were also transduced with GFP for control. Using this method, we were able to achieve approximately a 10‐fold overexpression of MEK1 protein in 9609 cells in both the cytoplasm and EV compartment (Figure 2a), suggesting that EV levels of MEK1 might reflect the levels in the cytoplasm. Since MEK1 signalling could promote the production of EVs (Hikita et al., 2022), we assessed the size, production level, and morphology of MEK1‐low and MEK1‐hi EVs. Figure S4A,B shows no difference in these parameters between MEK1‐low and MEK1‐hi EVs. We next performed proteomic analysis of MEK1‐low and MEK‐hi EVs and confirmed overexpression of MEK1 (Figure S5A) and found 64% of proteins shared between MEK1‐low and MEK‐hi EVs (Figure S5B). Due to the highly sensitive nature and very low detection limit of LC–MS/MS, this list includes proteins that are expressed at extremely low levels in EVs and likely are not even present in all EVs. So, we refined our analysis to include only the most abundantly expressed proteins within the EVs. Under these criteria, we found that 222 proteins (91%) were unchanged in MEK1‐low versus MEK1‐hi EVs (Figure S6), suggesting that the overexpression of MEK1 in EVs only minimally affects the levels of other EV proteins beyond MEK1. We did not see differences in antigen processing or presentation proteins, indicating that MEK1 did not influence the packaging of tumour antigens into EVs, as described previously (Hikita et al., 2022).

### 3.3 MEK1 overexpression in progressor tumours delays tumour growth via adaptive immunity and MEK1 activity

Since MEK1 is a proto‐oncogene, and inhibitors of MEK1 are standard‐of‐care for the treatment of certain cancers, we expected MEK1 overexpression in tumour cells might increase their growth rate. Strikingly, we found that in immunocompetent mice, 9609‐MEK1‐hi tumours grew significantly slower than 9609‐MEK1‐low tumours in WT (*p* < 0.01 Figure 2b) but not RAG1^−/−^ animals (Figure 2c), suggesting that the reduced growth of MEK1‐hi tumours requires the adaptive immune system. Similar results were obtained with 9614‐MEK1‐hi tumour cell lines (Figure S7a). Moreover, when the slow‐growing MEK1‐hi tumours were treated with MEK1 inhibitor PD98059 (Di Paola et al., 2010), tumour growth was increased by 130% in WT (*p* = 0.114 Figure 2d) but not RAG‐/‐ animals (Figure 2e). Notably, treatment with PD98059 reduced growth of both MEK1‐hi and MEK‐low tumours in vitro (Figure S7B), suggesting that the increased growth effect of MEK1 inhibition in mice acted on a host cell rather than the tumour cell and must have outweighed the anti‐proliferative effects of MEK1 inhibition on the tumour cell itself.

### 3.4 Secreted factors from MEK1‐hi tumours delay tumour growth via adaptive immunity

To determine the mechanism by which MEK1 overexpression in tumour cells inhibited their growth, we performed a contralateral flank experiment (Figure 2f). In this experimental setup, we transplanted an ‘instigator tumour’ (either MEK1‐hi or MEK1‐low) on one flank and then challenged the animal with the parent tumour on the other flank. We reasoned that secreted factors such as EVs produced by the instigator tumour (either MEK‐hi or MEK1‐low) could affect the growth of the tumour on the other flank. Indeed, we found that after 14 days, tumours grown with a contralateral MEK1‐hi tumour were, on average, 63% smaller in area than tumours grown with a contralateral MEK1‐low tumour (35.2 mm^2^ vs. 96 mm^2^, *p* < 0.05, Figure 2g). When we performed the same experiment in mice lacking adaptive immunity (RAG1^−/−^ mice), we saw no significant difference in the growth of the contralateral tumour (221 mm^2^ vs. 180.3 mm^2^ Figure 2h), confirming that a systemic factor derived from MEK1‐hi tumours exerts its anti‐tumour effect through adaptive immune cells.

### 3.5 MEK1‐hi EVs are sufficient to delay tumour growth via adaptive immunity

**FIGURE 3:**
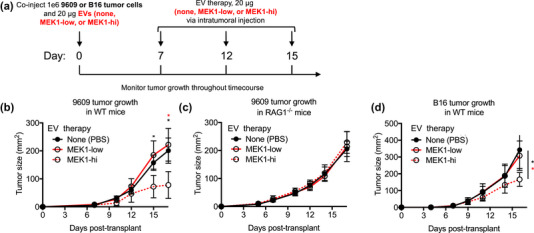
MEK1‐hi EVs are sufficient to delay tumour growth via adaptive immunity. (a) Schematic representation depicting workflow for experiment using MEK1‐low and MEK1‐hi EVs as therapeutic compounds against syngeneic 9609 or third party B16 tumours. 9609 or B16 tumour cells (1e6) were co‐transplanted with PBS (vehicle control), MEK1‐low, or MEK1‐hi EVs. Tumours were subsequently treated with PBS, MEK1‐low, or MEK1‐hi EVs via intratumoural injection on days 7, 12, and 15. Tumour growth was monitored over time. (b and c) Growth curves of 9609 tumours grown in (b) WT or (c) RAG1^‐/‐^ mice or (d) B16 tumours in WT mice treated with PBS, MEK1‐low, or MEK1‐hi EVs as described above Growth curve data are represented as mean ± SD. See also Figure S8.

Having demonstrated that a secreted factor from MEK1‐hi tumours has at least moderate anti‐tumour activity, we next tested the immune modulatory potential of MEK1‐hi versus MEK1‐low EVs. We co‐transplanted parental 9609 tumour cells with vehicle control (PBS), MEK1‐low, or MEK1‐hi EVs into WT or RAG1^−/−^ mice and monitored tumour growth. Over the 17‐day time course, we further treated tumours with vehicle control, MEK1‐low, or MEK1‐hi EVs via intratumoural injection on days 7, 12, and 15, in order to mimic the persistent release /presence of EVs from tumours (Figure 3a). We found that tumours treated with MEK1‐hi EVs were 61% and 55% smaller in area than tumours treated with vehicle control or with MEK1‐low EVs, respectively (*p* < 0.05 Figure 3b). This result translated in decreased weight as well (Figure S8a). When we performed the same experiment in RAG1^−/−^ mice, we found no significant difference in area or weight (Figures 3C, S8b) of tumours treated with vehicle control, MEK1‐low EVs, or MEK1‐hi EVs.

### 3.6 MEK1‐hi EVs delay 3rd party tumour growth and enhance efficacy of checkpoint blockade therapy

Since EVs could contain antigens and MHC (Andre et al., 2002, 2002; Koga et al., 2005; Raposo et al., 1996; Wolfers et al., 2001), we next tested the therapeutic effect of MEK1‐hi EVs on a third party tumour, using B16F10 as a model system. We found that B16F10 tumours treated with MEK1‐hi EVs (derived from 9609 fibrosarcoma cells) were 51% and 46% smaller in area (*p* < 0.05 Figure 3d) and weight (*p* < 0.1 Figure S8c) than tumours treated with vehicle control or MEK1‐low EVs, respectively. We did not find significant synergy when MEK1‐hi EVs were combined with anti‐PD1 therapy (Figure S8D and S9).

### 3.7 Myeloid cells uptake progressor EVs and contain EV‐delivered MEK1

**FIGURE 4:**
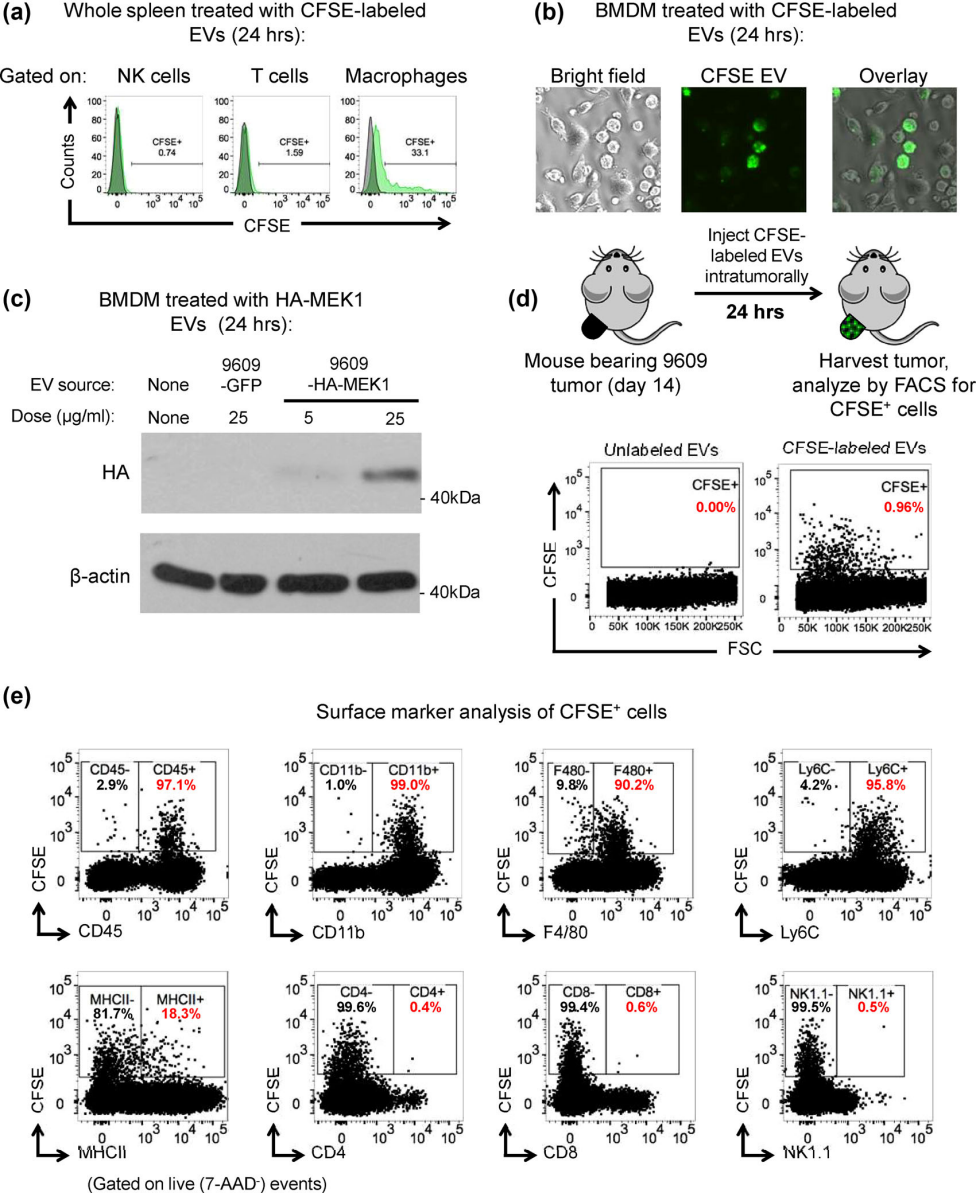
Myeloid cells uptake 9609 EVs and contain EV‐delivered MEK1. (a) Histograms showing CFSE expression in NK cells (CD45^+^, CD3^‐^, NK1.1^+^), T cells (CD45^+^, CD3^+^), and macrophages (CD45^+^, Ly6C^‐^, CD11b^+^, F4/80^+^) gated from whole spleen that was treated with unlabelled or CFSE‐labelled 9609 EVs for 24 h in vitro. Grey histograms represent cell treated with unlabelled EVs, and green histograms represent cells treated with CFSE‐labelled EVs. (b) Brightfield and fluorescent images of BMDM treated with CFSE‐labelled 9609 EVs for 24 h in vitro. (c) Western blot analysis showing HA and β‐actin expression in BMDM that were treated with HA‐MEK1‐containing 9609 EVs for 24 h in vitro. (d) Schematic representation depicting workflow to detect in vivo EV uptake, and dot plots showing CFSE expression in cells from single cell 9609 tumour suspensions that were treated with 20 µg of unlabelled or CFSE‐labelled 9609 EVs for 24 h. (e) Dot plots showing expression of CFSE vs. CD45, CD11b, F4/80, Ly6C, MHCII, CD4, CD8, and NK1.1 in single cell suspensions of 9609 tumours treated with 20 µg of CFSE‐labelled 9609 EVs.

To determine which cell types can uptake 9609 EVs, we administered CFSE labelled EVs (Pospichalova et al., 2015) to splenocytes and found that NK cells and T cells had low to no CFSE signal, whereas about 30% of macrophages were CFSE^+^ (Figure 4a). The EVs were taken inside macrophages as shown by microscopy (Figure 4b).

To determine if EV‐MEK1 could be transferred to the cytoplasm of macrophages that uptake EVs, we generated a cell line expressing HA‐tagged MEK1 in both the cytoplasm and EV compartments (Figure S10). Next, we treated BMDM with vehicle control (PBS), GFP EVs, or HA‐MEK1 EVs and found a dose‐dependent increase in HA signal in the cytoplasm of BMDM that were treated with HA‐MEK1 EVs (Figure 4c) demonstrating that proteins within the EVs, and specifically MEK1, are transferred to the cytoplasm of the target cell.

We next sought to determine if we could observe and quantify EV uptake in vivo. To this end, we injected unlabelled or CFSE‐labelled EVs into established, day 14 9609 tumours via intratumoural injection. After 24 h, we harvested the tumours, created a single‐cell suspension, and analysed these cells for CFSE expression (Figure 4d). We found no CFSE signal in tumours injected with unlabelled EVs. In tumours injected with CFSE‐labelled EVs, almost 1% of all cells in the single‐cell suspension were CFSE^+^. Notably, the vast majority of CFSE^+^ cells expressed CD45 (97%), CD11b (99%), F4/80 (90.2%), Ly6C (95.8%), and to a lesser degree, MHCII (18.3%) (Figure 4e). Conversely, we found little to no expression of CD4, CD8, or NK1.1 on cells expressing CFSE, confirming that mostly tumour‐associated macrophages (CD11b^+^ F4/80^+^, 90% of CFSE+ cells) take up EVs in vivo.

### 3.8 MEK1‐hi EVs promote the accumulation of anti‐tumour immune effector cells

**FIGURE 5:**
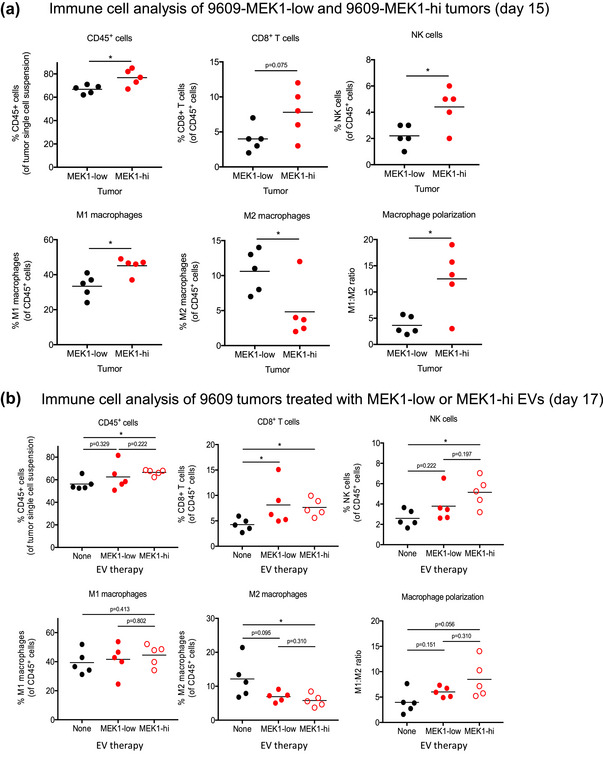
MEK1‐hi EVs boost anti‐tumour immunity. (a) Quantification of relative frequencies of CD45^+^ cells, CD8^+^ T cells (CD45^+^, CD3^+^, CD8^+^, CD4^‐^), NK cells (CD45^+^, CD3^‐^, NK1.1^+^), M1‐type macrophages (CD45^+^, Ly6C^‐^, F4/80^+^, MHCII^hi^) and M2‐type macrophages (CD45^+^, Ly6C^‐^, F4/80^+^, MHCII^low^), and M1:M2 ratios in 9609‐MEK1‐low or 9609‐MEK1‐hi tumours grown in WT mice for 15 days. (b) Quantification of relative frequencies of CD45^+^ cells, CD8^+^ T cells, NK cells, M1‐type macrophages, and M2‐type macrophages, and M1:M2 ratios in 9609 tumours grown in WT mice and treated with EVs at days 0, 7, 11, and 15 for 17 days. *n* = 5 mice per group. Data are plotted as individual values, with each symbol representing an individual tumour, and the horizontal line represents the mean of each group.

To gain further insight into the mechanisms mediating the anti‐tumour effect of MEK1‐hi EVs, we analysed the infiltrating immune cells in 9609‐MEK1‐low and 9609‐MEK1‐hi tumours (Figure 5a). We found that compared to MEK1‐low tumours, MEK1‐hi tumours had a 15% increase in the relative frequency of CD45^+^ cells (*p* < 0.05), a 95% increase in CD8^+^ T cells (*p* = 0.075), and a 100% increase in NK cells (*p* < 0.05). We also observed a striking shift toward anti‐tumour macrophages (MHC class II high, CD206 low, “M1‐type”) in MEK1‐hi compared to MEK1‐low tumours. Specifically, we saw that anti‐tumour macrophages were increased by 35% (*p* < 0.05) and pro‐tumour (MHC class II low CD206 hi “M2‐type”) macrophages were decreased by 55% (*p* < 0.05) in MEK1‐hi compared to MEK1‐low tumours, manifesting in a 247% increase in the ratio of these macrophages (*p* < 0.05).

Since the changes in immune cells described above cannot be definitively attributed to EVs, we repeated the experiment but with 9609 tumours that were co‐injected and treated with vehicle control, MEK1‐low EVs, or MEK1‐hi EVs. We did not find statistically significant results, presumably because the exposure of these cells to EVs was intermittent rather than continuous, but the trends did mirror those from the previous experiment, including increased CD8^+^ T cells, increased NK cells, increased anti‐tumour and decreased pro‐tumour macrophages within the tumour (Figure 5b).

### 3.9 Gene expression studies demonstrate that macrophages exposed to EV‐MEK1 display an IFNγ signature that is important for anti‐tumour effects

**FIGURE 6:**
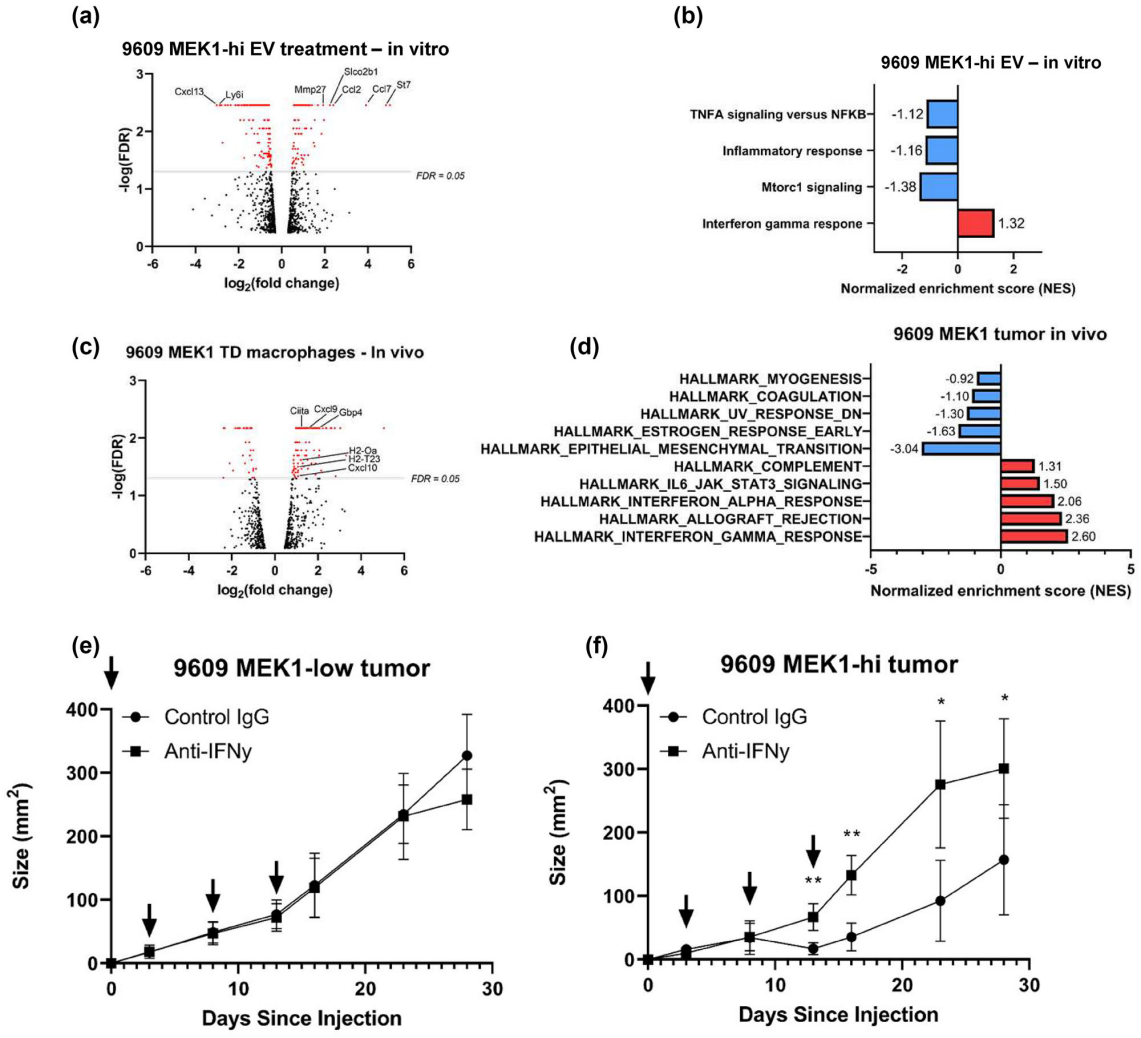
MEK1‐hi EVs induce pro‐inflammatory chemokines and IFNγ response genes in tumour‐associated macrophages. (A and C) Volcano plots showing significantly upregulated gene expression of (A) 9609 tumour macrophages treated with 9609 MEK1‐hi EVs in vitro and (C) 9609 MEK1‐hi tumour macrophages in vivo (*p* < 0.05, FDR < 0.05). (B and D) Gene set enrichment analysis (version 4.1.0) was performed on differentially expressed gene lists obtained from (A) and (C). Comparison was against (B) treatment with 9609 MEK1‐low EVs and (D) 9609 MEK1‐low tumour macrophages. On day 0, (E) 9609 MEK1‐low or (F) 9609 MEK1‐hi cells were subcutaneously injected into mice and 250 µg of either anti‐IFNγ or control IgG was IP injected (shown by arrows). Tumour measurement is length × width (mm^2^). Each plotted point is an average measurement of 9‐11 mice. * Denotes *p* < 0.01, ** *p* < 0.0001 using a Student's *t* test. Error bars represent standard deviation. See also Figure S11 and Table S2.

We next examined the gene expression profile of macrophages exposed to MEK1‐hi versus MEK1‐low EVs in vitro or in vivo. Figure 6 shows the volcano plot of differentially expressed genes (Figure 6a,c) and gene set enrichment analysis (GSEA) (Subramanian et al., 2005) (Figure 6b,d) of tumour‐derived macrophages exposed to MEK1‐hi versus MEK1‐low EVs in vitro (Figure 6a,b) or in vivo (Figure 6c,d). Notably, in vitro exposure induced the expression of chemokines such as CCL2 and CCL7, whereas in vivo exposure induced the expression of mostly IFNγ‐related genes involved in antigen presentation (CIITA, H2) and IFNγ‐upregulated chemokines (CXCL9, CXCL10) (Figures 6a,c,S11). GSEA analysis confirmed the association of MEK‐1‐hi EV exposure with IFNγ‐induced gene signatures (Figure 6b–d). To demonstrate the functional impact of the observed IFNγ signature, we treated mice bearing MEK1‐low and MEK1‐hi 9609 tumours with control IgG or antibodies blocking IFNγ. Figure 6e,f shows that anti‐IFNγ but not control IgG increased the growth of 9609‐MEK1‐hi (*p* < 0.01) but not 9609 MEK1‐low tumours, suggesting that EV‐MEK1 uptaken by macrophages induced the recruitment of IFNγ‐producing lymphocytes via induction of chemokines, resulting in the in vivo exposure of TD macrophages to functional IFNγ. The source of IFNγ is likely T cells, as depletion of NK cells did not restore growth of 9609‐MEK1‐hi tumours (Figure S12). We were not able to specifically deplete macrophages that might take up ECVs, but whole body‐depletion of monocytes/macrophages did not increase tumour growth (Figure S12), suggesting that the subset of macrophages that promotes tumour rejection might be counterbalanced by macrophages that promote tumour growth, and depleting both had minimal effects on tumour growth.

## 4 DISCUSSION

We have observed that overexpression of MEK1 in progressor tumour cell lines paradoxically reduces their growth in an immune‐dependent mechanism. This reduction in growth is partly attributable to increased MEK1 in EVs and is correlated with increased immune cell activity in MEK1‐overexpressing tumours. These results are surprising since MEK1 is an oncogene that should promote tumour growth. Moreover, previous studies found that knocking out MEK1 in tumour cells can induce immunogenic antigen presentation genes (Dennison et al., 2021) and MEK1 inhibitors can promote immune responses (Baumann et al., 2020; Prasad et al., 2022). Thus, we had anticipated that tumour‐suppressive proteins would be enriched in regressor cell lines and their EVs. Our discovery suggests that the role of MEK1 in cancer could have context‐dependent opposing functions in cell‐intrinsic versus cell‐extrinsic activities. Cell‐intrinsic MEK1 can act as an oncogene, inducing cellular transformation and promoting solid tumour growth (Cowley et al., 1994; Mansour et al., 1994). In contrast, EV‐MEK1 acts as a tumour suppressor by stimulating anti‐tumour immunity via a mechanism likely involving macrophages, IFNγ, and adaptive immunity. Notably, expression of MEK1 in tumours from patients also has context‐dependent impact on prognosis (Zhou et al., 2021). For example, in this pan‐cancer analysis (Zhou et al., 2021), high MEK1 mRNA in lung, renal, and liver cancer harboured a worse overall survival. On the other hand, high MEK1 mRNA in thymoma, stomach cancer, and head and neck cancer correlated with better overall survival. Interestingly, at the protein level, high MEK1 expression only correlated with good prognosis in certain tumours but did not correlate with bad prognosis. It would be interesting to examine whether the MEK1 content in tumour‐derived EVs of cancer patients would determine the impact of tumour MEK1 expression on prognosis. On the other hand, given that MEK1 in EVs may only partially explain the beneficial effect of MEK1 overexpression, further studies on downstream gene expression changes in 9609‐MEK1 versus 9609‐GFP could elucidate other mechanisms by which the tumour suppressor MEK1 might counterintuitively activate the immune system.

We found that uptake of EV‐MEK1 by macrophages results in gene expression changes that promote recruitment of IFNγ‐producing lymphocytes, leading to increased antigen presentation and activation of adaptive immunity. Immune stimulatory roles for EVs include delivery of antigens to immune cells (Andre et al., 2002, 2002; Koga et al., 2005; Wolfers et al., 2001) and activation of patrolling monocytes (Plebanek et al., 2017). Our studies add another mechanism by which EVs might stimulate immunity—via activation of intratumoural macrophages. These results support the further investigation of delivery of MEK1 to macrophages as a form of cancer therapy while also developing better targeting of MEK1 inhibitors to specifically inhibit MEK1 in tumours while sparing MEK1 activity in immune cells such as macrophages.

As a highly plastic and heterogeneous cell lineage, macrophages represent a key population for therapeutic targeting in cancer (Cassetta & Pollard, 2018). Our findings provide an important intracellular signalling target that could promote macrophage anti‐tumour responses. Multiple intracellular signalling pathways can dictate macrophage responses (Zhou et al., 2014). For example, PI3Kγ signalling in macrophages promotes immune suppression and tumour growth via Akt‐ and mTor‐mediated inhibition of NFκB and activation of C/EBPβ (Kaneda et al., 2016; Vergadi et al., 2017). In contrast Notch, JAK/STAT, and TLR signalling can promote macrophage activation, resulting in tumour inhibition (Hu et al., 2007; Wang et al., 2010). Our findings highlight the MEK1/MAP kinase pathway for agonist targeting to improve anti‐tumour immunity.

Indeed, other studies have found that MAP kinase activation could promote anti‐tumour macrophages similar to IFNγ‐activated M1‐type macrophages. Specifically, TSC1 was shown to inhibit M1‐type polarization via inhibition of the MAP kinase cascade (Zhu et al., 2014), whereas MEK1/2 inhibition could promote IL‐5 production (Li et al., 2016) and reparative properties of macrophages (Long et al., 2017), resulting in reduced lung inflammation (Kurian et al., 2019; Long et al., 2017). In contrast, other studies have found that MEK1/2 inhibition selectively eliminated M2‐type macrophages using a model system which had sparse M1‐type macrophages and tumour cells that underwent immunogenic cell death due to MEK1/2 inhibition (Baumann et al., 2021). Finally, while our studies support the paradigm that MEK1 activation in macrophages promotes IFNγ‐dependent responses, another study found that MEK1 inhibition actually restored responsiveness to IFNγ (Yang & Ding, 2019). Clearly, the context of MEK1 activation in macrophages will critically impact how to best deploy therapies targeting this pathway for cancer treatment. This context‐dependent activity of MEK1 could explain why global inhibition of MEK1 in our experiments (Figure 2d) showed a trend for increased growth but lacked statistical significance.

We found that MEK1‐hi EVs provided only slight additional benefit to anti‐PD1 therapy against B16 melanoma. Interestingly, combining MEK1 inhibitors with checkpoint blockade has resulted in differing effects. In melanoma, MEK inhibitors can actually augment the efficacy of checkpoint blockade (Hu‐Lieskovan et al., 2014). In contrast, when combined with anti‐PDL1 in colorectal cancer, MEK1 inhibition did not provide any benefit (Eng et al., 2019; Hellmann et al., 2019). These disparate results could reflect the fact that MAP kinase pathway inhibition could upregulate tumour‐associated antigens and promote T cell infiltration and production of pro‐inflammatory cytokines via immunogenic cell death (Brea et al., 2016; Cooper et al., 2013, 2013; Frederick et al., 2013), resulting in additive or synergistic effects. On the contrary, MEK1 inhibition could also have immune suppressive effects, as suggested by our data. This previously unappreciated effect of MEK1 inhibitors provides important inroads into the rational design of clinical trials and MEK1‐based drugs. For example, in patients whose tumours have high MEK1 expression in EVs, MEK1 inhibition could prevent the anti‐tumour effect of these EVs, thus actually promoting tumour growth instead of inhibiting it. Our results therefore suggest that screening cancer patient EVs for the presence of MEK1 could be a useful form of “personalized medicine” to select patient populations who most likely would respond to MEK inhibitors without the deleterious effects caused by inhibiting MEK1 activity in EVs.

## AUTHOR CONTRIBUTIONS

**Stephen C. Searles**: Conceptualization (lead); data curation (lead); formal analysis (lead); investigation (lead); methodology (lead); writing—original draft (lead); writing—review and editing (supporting). **Wei‐Shan Chen**: Data curation (supporting); formal analysis (supporting). **Jarrod D. Yee**: Data curation (supporting); methodology (supporting). **Preston Lee**: Data curation (supporting). **Calvin K. Lee**: Data curation (supporting); investigation (supporting); methodology (supporting). **Christine Caron**: Data curation (supporting); methodology (supporting); project administration (supporting); writing—review and editing (supporting). **Felippe Neto**: Data curation (supporting). **Irina Matei**: Conceptualization (supporting); data curation (supporting); methodology (supporting); writing—review and editing (supporting). **David Lyden**: Conceptualization (supporting); funding acquisition (supporting); project administration (supporting); writing—review and editing (supporting). **Jack D. Bui**: Conceptualization (supporting); data curation (supporting); formal analysis (supporting); funding acquisition (lead); investigation (supporting); methodology (supporting); project administration (lead); resources (lead); supervision (lead); validation (supporting); writing—original draft (supporting); writing—review and editing (lead).

## CONFLICT OF INTEREST STATEMENT

The authors declare no conflicts of interest.

## Supporting information



Supporting information

Supporting information

Supporting information
