# Supplementary material for: MAP kinase kinase 1 (MEK1) within extracellular vesicles inhibits tumour growth by promoting anti‐tumour immunity
Source: J Extracell Vesicles. 2024 Sep 27;13(10):e12515. doi: 10.1002/jev2.12515 (PMC11428867; doi:10.1002/jev2.12515)
Supplement: Supplementary file 1 — Supporting information [file JEV2-13-e12515-s002.pptx]

## Slide 1
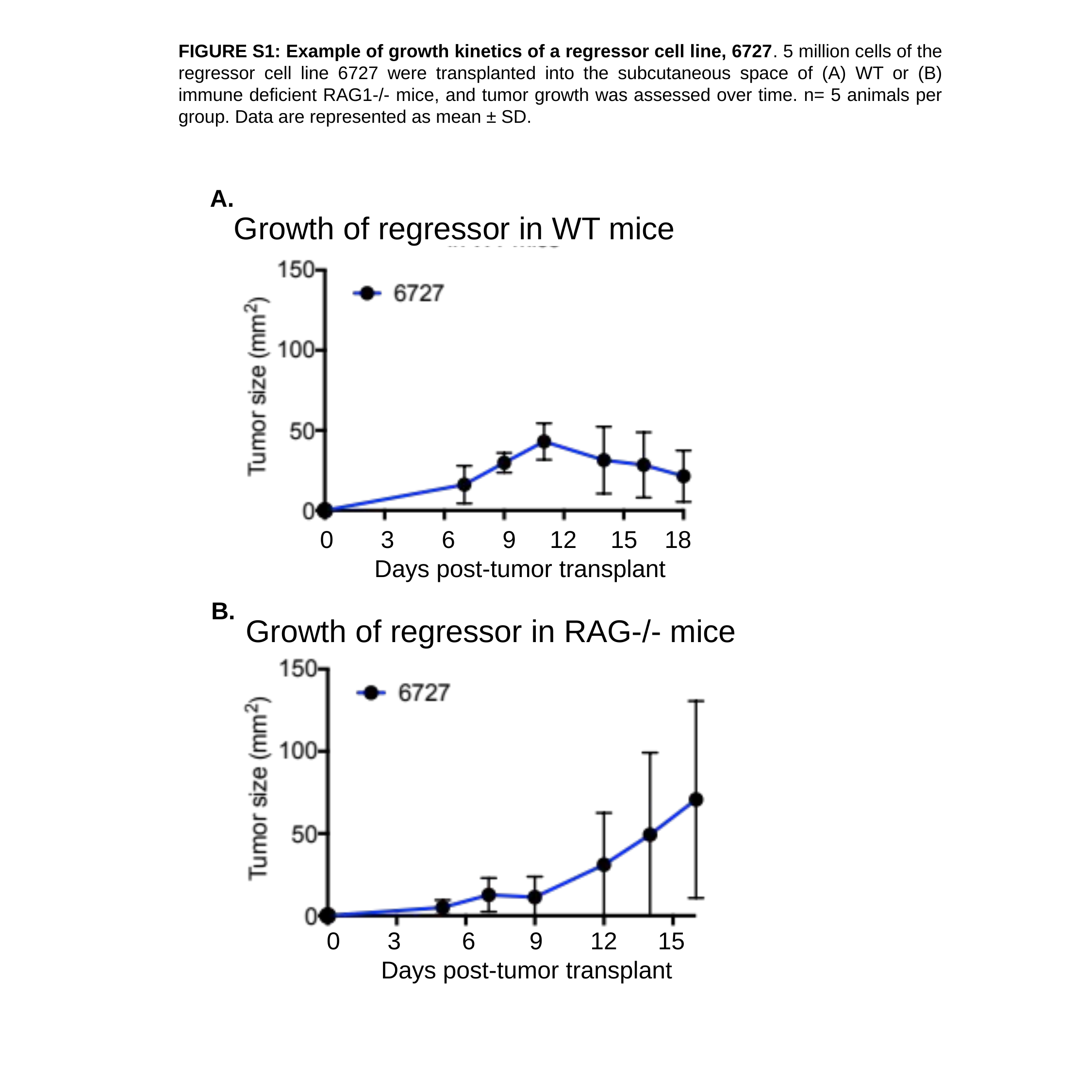

FIGURE S1: Example of growth kinetics of a regressor cell line, 6727. 5 million cells of the regressor cell line 6727 were transplanted into the subcutaneous space of (A) WT or (B) immune deficient RAG1-/- mice, and tumor growth was assessed over time. n= 5 animals per group. Data are represented as mean ± SD.
A.
Growth of regressor in WT mice
0 3 6 9 12 15 18
 Days post-tumor transplant
B.
Growth of regressor in RAG-/- mice
0 3 6 9 12 15
 Days post-tumor transplant

## Slide 2
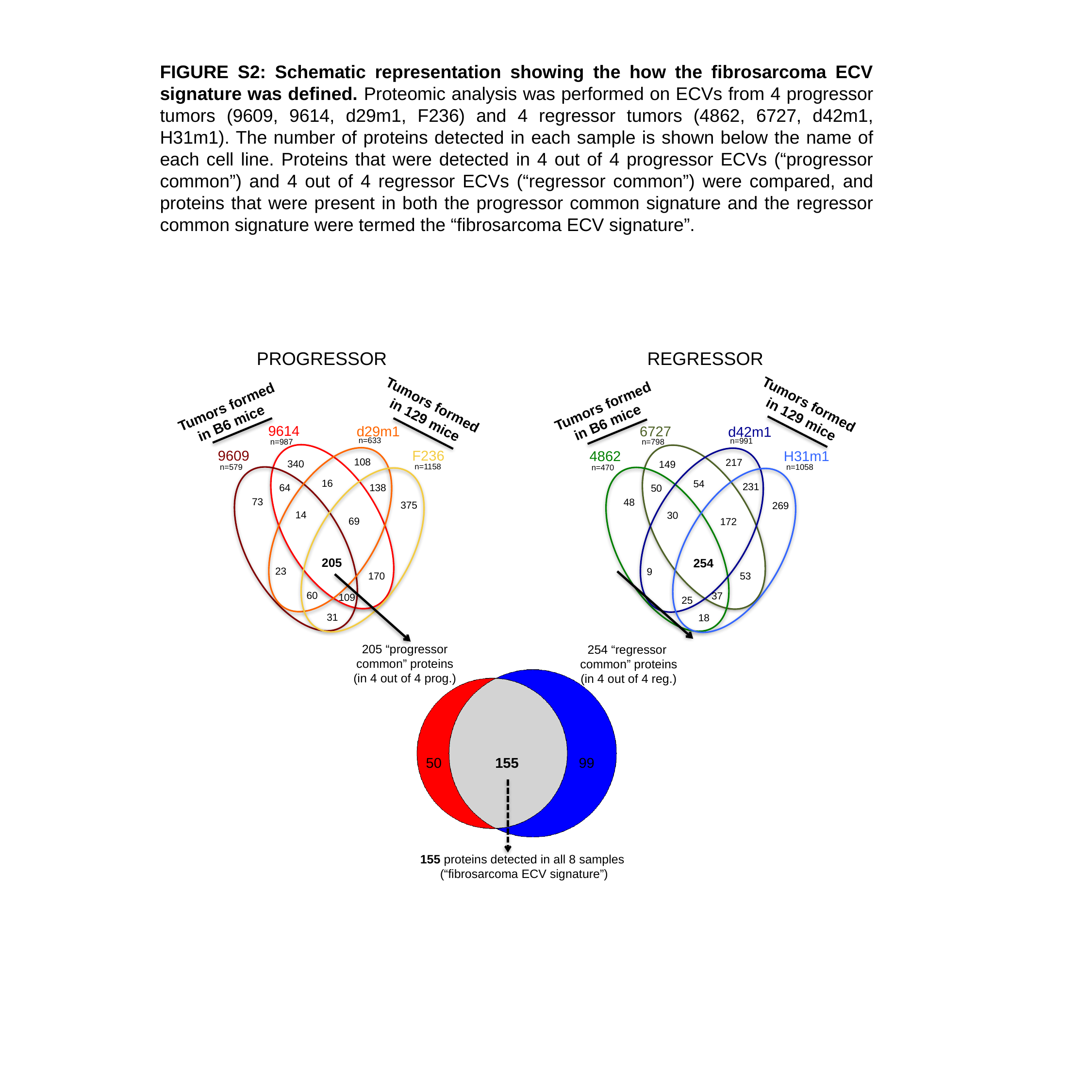

FIGURE S2: Schematic representation showing the how the fibrosarcoma ECV signature was defined. Proteomic analysis was performed on ECVs from 4 progressor tumors (9609, 9614, d29m1, F236) and 4 regressor tumors (4862, 6727, d42m1, H31m1). The number of proteins detected in each sample is shown below the name of each cell line. Proteins that were detected in 4 out of 4 progressor ECVs (“progressor common”) and 4 out of 4 regressor ECVs (“regressor common”) were compared, and proteins that were present in both the progressor common signature and the regressor common signature were termed the “fibrosarcoma ECV signature”.
PROGRESSOR
REGRESSOR
Tumors formed in 129 mice
Tumors formed in 129 mice
Tumors formed in B6 mice
Tumors formed in B6 mice
9614
d29m1
n=633
n=987
64
138
205
9609
F236
108
340
n=1158
n=579
16
73
375
14
69
23
170
60
109
31
6727
d42m1
n=991
n=798
231
50
254
4862
H31m1
217
149
n=1058
n=470
54
48
269
30
172
9
53
37
25
18
205 “progressor common” proteins
(in 4 out of 4 prog.)
254 “regressor
common” proteins
(in 4 out of 4 reg.)
50
155
99
155 proteins detected in all 8 samples
 (“fibrosarcoma ECV signature”)

## Slide 3
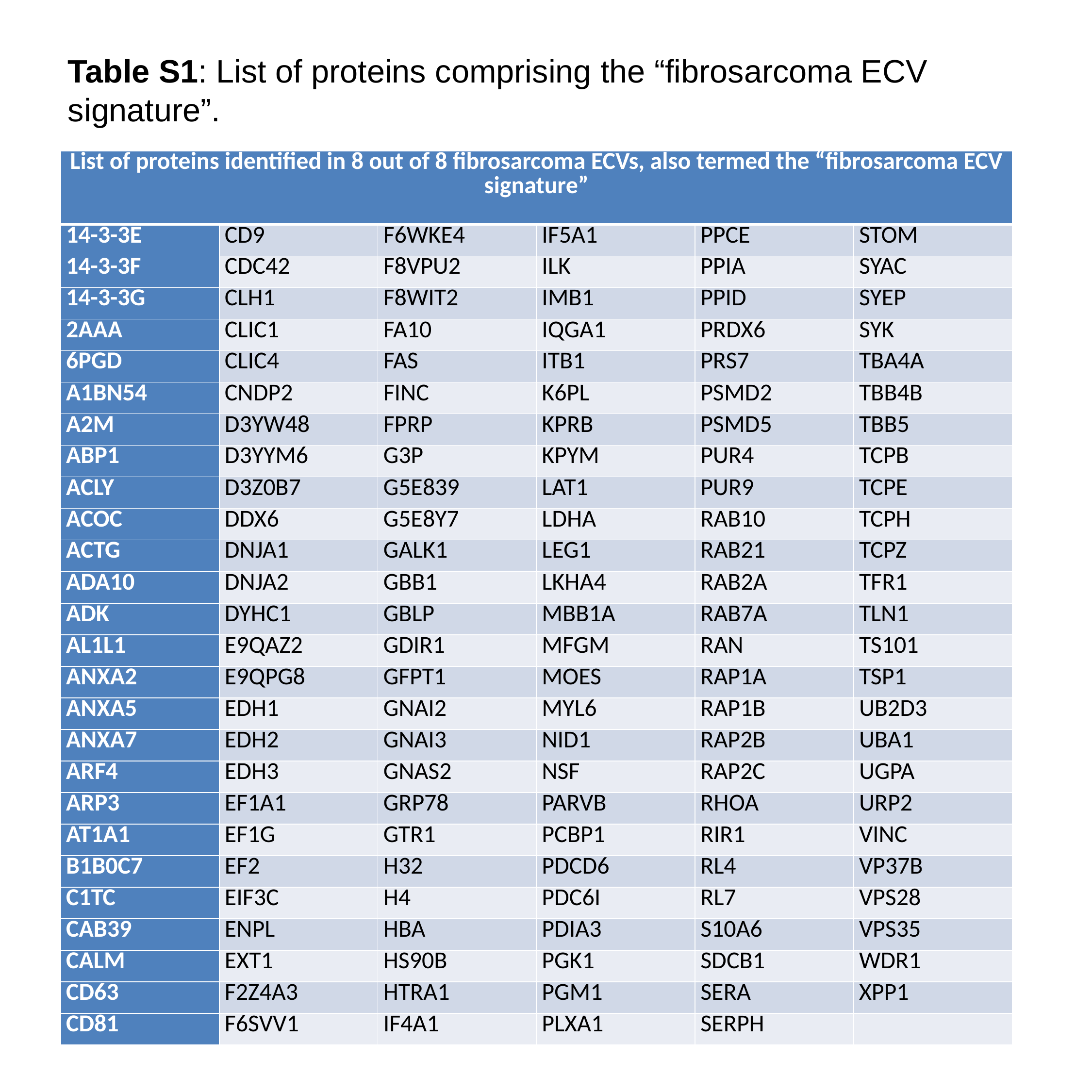

Table S1: List of proteins comprising the “fibrosarcoma ECV signature”.
| List of proteins identified in 8 out of 8 fibrosarcoma ECVs, also termed the “fibrosarcoma ECV signature” | | | | | |
| --- | --- | --- | --- | --- | --- |
| 14-3-3E | CD9 | F6WKE4 | IF5A1 | PPCE | STOM |
| 14-3-3F | CDC42 | F8VPU2 | ILK | PPIA | SYAC |
| 14-3-3G | CLH1 | F8WIT2 | IMB1 | PPID | SYEP |
| 2AAA | CLIC1 | FA10 | IQGA1 | PRDX6 | SYK |
| 6PGD | CLIC4 | FAS | ITB1 | PRS7 | TBA4A |
| A1BN54 | CNDP2 | FINC | K6PL | PSMD2 | TBB4B |
| A2M | D3YW48 | FPRP | KPRB | PSMD5 | TBB5 |
| ABP1 | D3YYM6 | G3P | KPYM | PUR4 | TCPB |
| ACLY | D3Z0B7 | G5E839 | LAT1 | PUR9 | TCPE |
| ACOC | DDX6 | G5E8Y7 | LDHA | RAB10 | TCPH |
| ACTG | DNJA1 | GALK1 | LEG1 | RAB21 | TCPZ |
| ADA10 | DNJA2 | GBB1 | LKHA4 | RAB2A | TFR1 |
| ADK | DYHC1 | GBLP | MBB1A | RAB7A | TLN1 |
| AL1L1 | E9QAZ2 | GDIR1 | MFGM | RAN | TS101 |
| ANXA2 | E9QPG8 | GFPT1 | MOES | RAP1A | TSP1 |
| ANXA5 | EDH1 | GNAI2 | MYL6 | RAP1B | UB2D3 |
| ANXA7 | EDH2 | GNAI3 | NID1 | RAP2B | UBA1 |
| ARF4 | EDH3 | GNAS2 | NSF | RAP2C | UGPA |
| ARP3 | EF1A1 | GRP78 | PARVB | RHOA | URP2 |
| AT1A1 | EF1G | GTR1 | PCBP1 | RIR1 | VINC |
| B1B0C7 | EF2 | H32 | PDCD6 | RL4 | VP37B |
| C1TC | EIF3C | H4 | PDC6I | RL7 | VPS28 |
| CAB39 | ENPL | HBA | PDIA3 | S10A6 | VPS35 |
| CALM | EXT1 | HS90B | PGK1 | SDCB1 | WDR1 |
| CD63 | F2Z4A3 | HTRA1 | PGM1 | SERA | XPP1 |
| CD81 | F6SVV1 | IF4A1 | PLXA1 | SERPH | |

## Slide 4
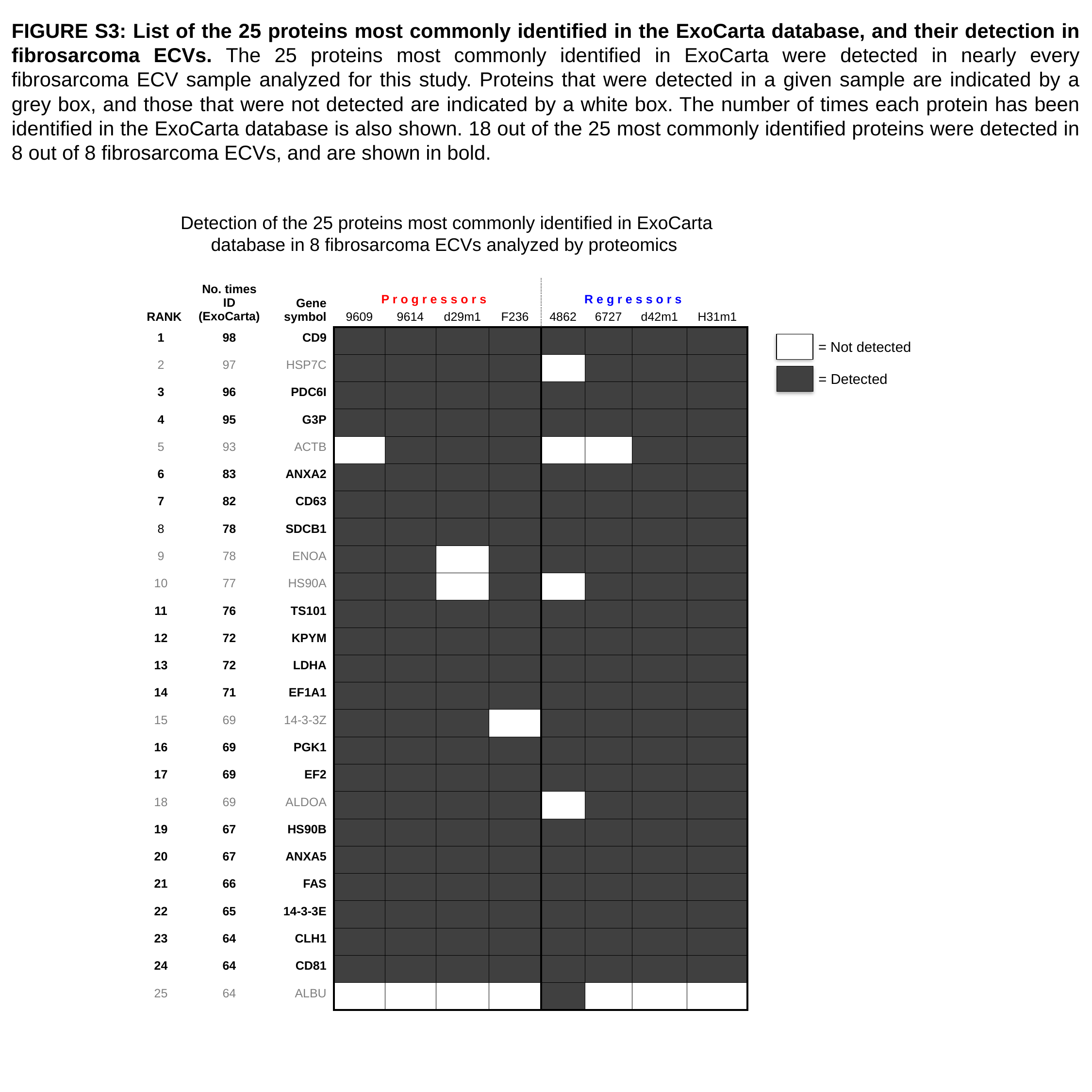

FIGURE S3: List of the 25 proteins most commonly identified in the ExoCarta database, and their detection in fibrosarcoma ECVs. The 25 proteins most commonly identified in ExoCarta were detected in nearly every fibrosarcoma ECV sample analyzed for this study. Proteins that were detected in a given sample are indicated by a grey box, and those that were not detected are indicated by a white box. The number of times each protein has been identified in the ExoCarta database is also shown. 18 out of the 25 most commonly identified proteins were detected in 8 out of 8 fibrosarcoma ECVs, and are shown in bold.
Detection of the 25 proteins most commonly identified in ExoCarta database in 8 fibrosarcoma ECVs analyzed by proteomics
| | | | | | | | | | | |
| --- | --- | --- | --- | --- | --- | --- | --- | --- | --- | --- |
| RANK | No. times ID (ExoCarta) | Gene symbol | 9609 | 9614 | d29m1 | F236 | 4862 | 6727 | d42m1 | H31m1 |
| 1 | 98 | CD9 | | | | | | | | |
| 2 | 97 | HSP7C | | | | | | | | |
| 3 | 96 | PDC6I | | | | | | | | |
| 4 | 95 | G3P | | | | | | | | |
| 5 | 93 | ACTB | | | | | | | | |
| 6 | 83 | ANXA2 | | | | | | | | |
| 7 | 82 | CD63 | | | | | | | | |
| 8 | 78 | SDCB1 | | | | | | | | |
| 9 | 78 | ENOA | | | | | | | | |
| 10 | 77 | HS90A | | | | | | | | |
| 11 | 76 | TS101 | | | | | | | | |
| 12 | 72 | KPYM | | | | | | | | |
| 13 | 72 | LDHA | | | | | | | | |
| 14 | 71 | EF1A1 | | | | | | | | |
| 15 | 69 | 14-3-3Z | | | | | | | | |
| 16 | 69 | PGK1 | | | | | | | | |
| 17 | 69 | EF2 | | | | | | | | |
| 18 | 69 | ALDOA | | | | | | | | |
| 19 | 67 | HS90B | | | | | | | | |
| 20 | 67 | ANXA5 | | | | | | | | |
| 21 | 66 | FAS | | | | | | | | |
| 22 | 65 | 14-3-3E | | | | | | | | |
| 23 | 64 | CLH1 | | | | | | | | |
| 24 | 64 | CD81 | | | | | | | | |
| 25 | 64 | ALBU | | | | | | | | |
P r o g r e s s o r s
R e g r e s s o r s
= Not detected
= Detected

## Slide 5
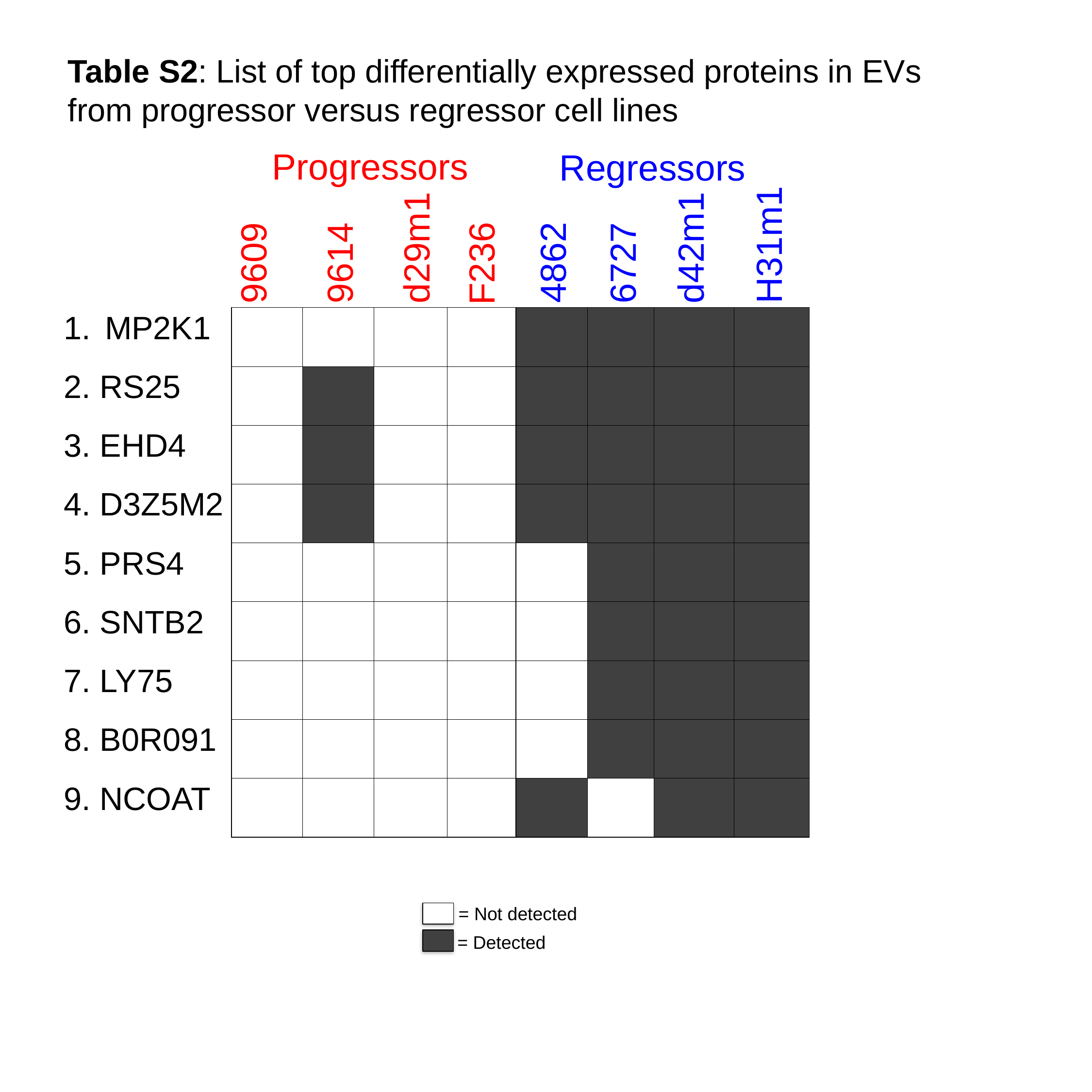

Table S2: List of top differentially expressed proteins in EVs from progressor versus regressor cell lines
Progressors
Regressors
d42m1
d29m1
H31m1
4862
9609
9614
6727
F236
| MP2K1 |
| --- |
| 2. RS25 |
| 3. EHD4 |
| 4. D3Z5M2 |
| 5. PRS4 |
| 6. SNTB2 |
| 7. LY75 |
| 8. B0R091 |
| 9. NCOAT |
| | | | | | | | |
| --- | --- | --- | --- | --- | --- | --- | --- |
| | | | | | | | |
| | | | | | | | |
| | | | | | | | |
| | | | | | | | |
| | | | | | | | |
| | | | | | | | |
| | | | | | | | |
| | | | | | | | |
= Not detected
= Detected

## Slide 6
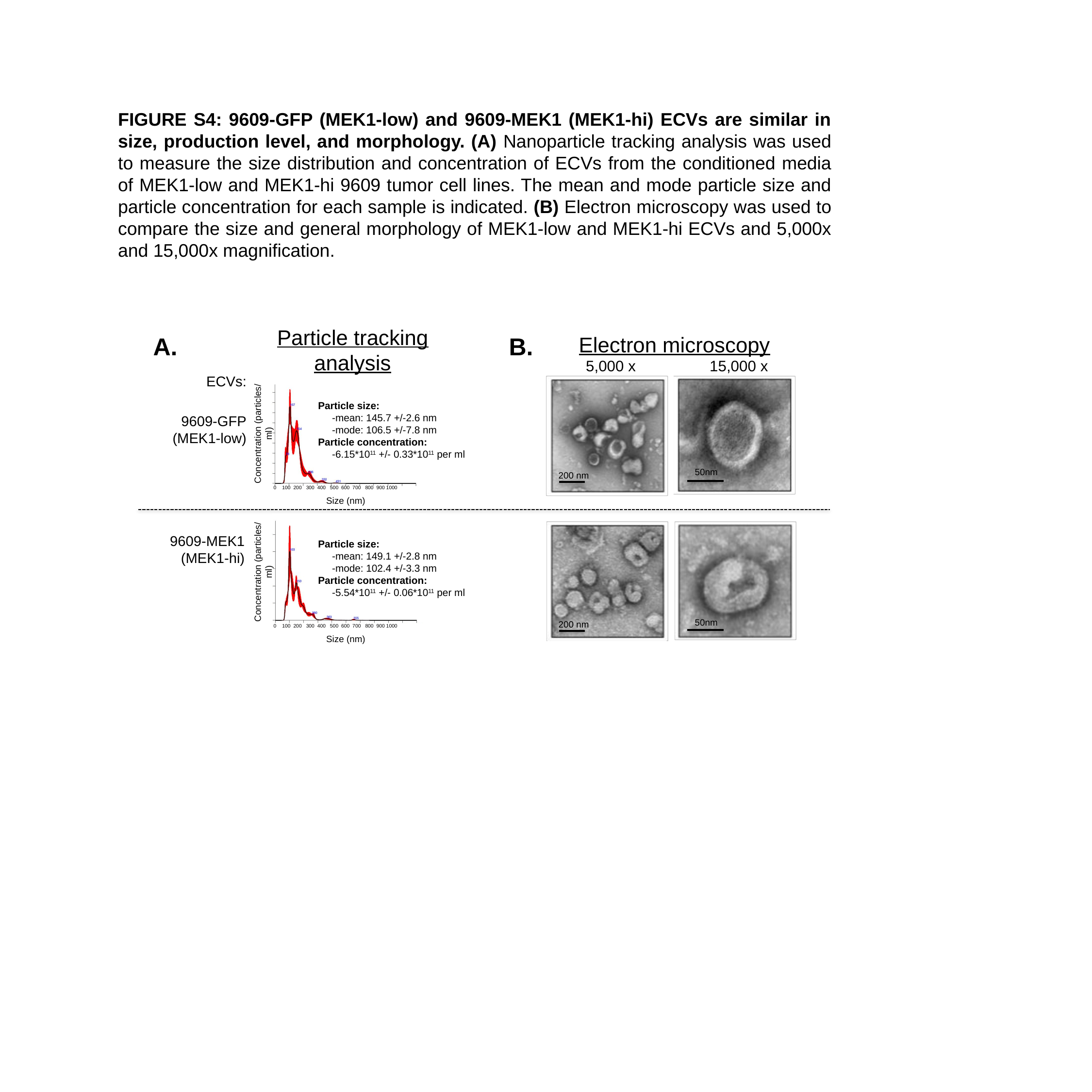

FIGURE S4: 9609-GFP (MEK1-low) and 9609-MEK1 (MEK1-hi) ECVs are similar in size, production level, and morphology. (A) Nanoparticle tracking analysis was used to measure the size distribution and concentration of ECVs from the conditioned media of MEK1-low and MEK1-hi 9609 tumor cell lines. The mean and mode particle size and particle concentration for each sample is indicated. (B) Electron microscopy was used to compare the size and general morphology of MEK1-low and MEK1-hi ECVs and 5,000x and 15,000x magnification.
Particle tracking analysis
A.
B.
Electron microscopy
5,000 x
15,000 x
50nm
200 nm
50nm
200 nm
ECVs:
Concentration (particles/ ml)
0 100 200 300 400 500 600 700 800 900 1000
Size (nm)
Particle size:
 -mean: 145.7 +/-2.6 nm
 -mode: 106.5 +/-7.8 nm
Particle concentration:
 -6.15*1011 +/- 0.33*1011 per ml
9609-GFP
(MEK1-low)
Concentration (particles/ ml)
0 100 200 300 400 500 600 700 800 900 1000
Size (nm)
Particle size:
 -mean: 149.1 +/-2.8 nm
 -mode: 102.4 +/-3.3 nm
Particle concentration:
 -5.54*1011 +/- 0.06*1011 per ml
9609-MEK1 (MEK1-hi)

## Slide 7
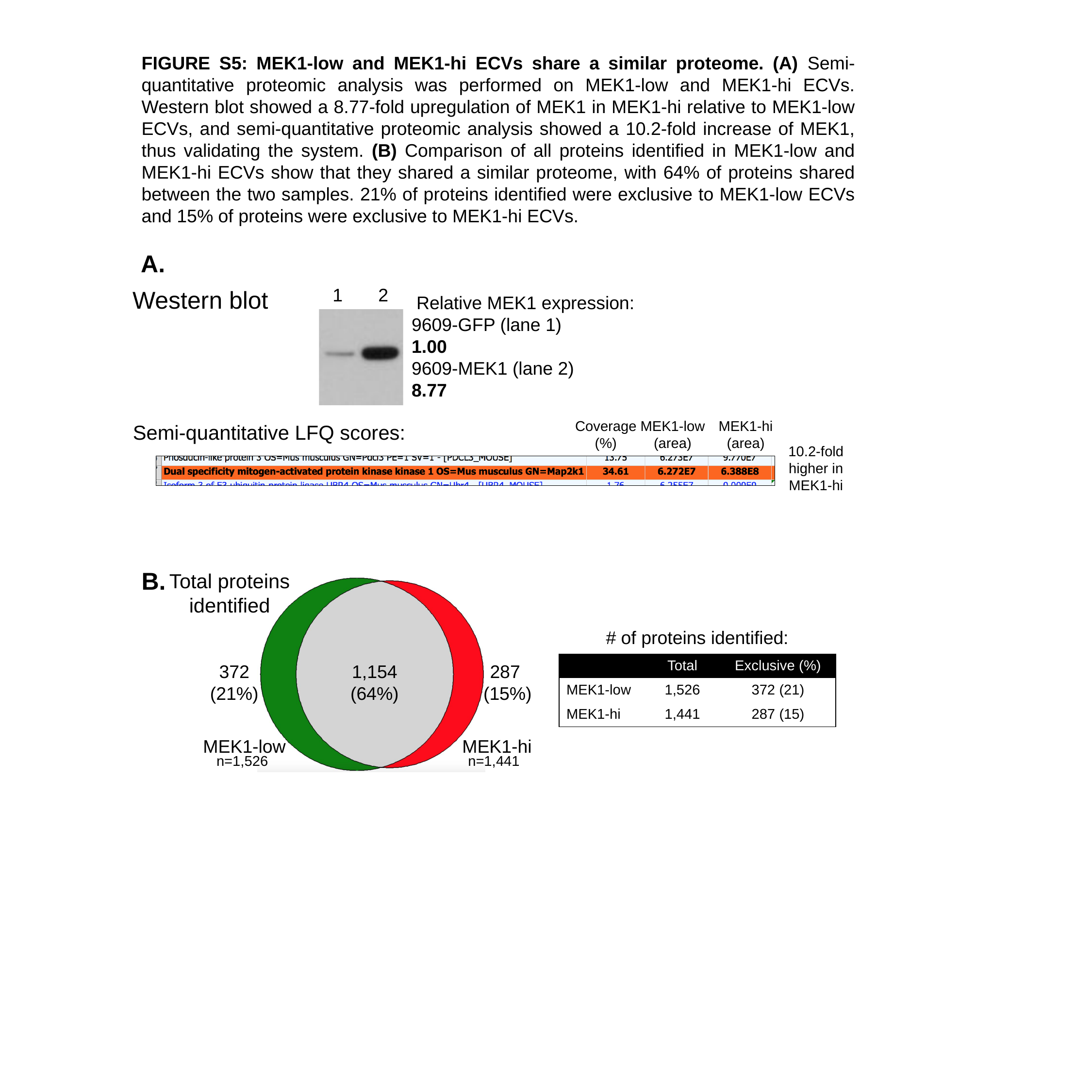

FIGURE S5: MEK1-low and MEK1-hi ECVs share a similar proteome. (A) Semi-quantitative proteomic analysis was performed on MEK1-low and MEK1-hi ECVs. Western blot showed a 8.77-fold upregulation of MEK1 in MEK1-hi relative to MEK1-low ECVs, and semi-quantitative proteomic analysis showed a 10.2-fold increase of MEK1, thus validating the system. (B) Comparison of all proteins identified in MEK1-low and MEK1-hi ECVs show that they shared a similar proteome, with 64% of proteins shared between the two samples. 21% of proteins identified were exclusive to MEK1-low ECVs and 15% of proteins were exclusive to MEK1-hi ECVs.
A.
1 2
Relative MEK1 expression:
9609-GFP (lane 1) 	 1.00
9609-MEK1 (lane 2) 	 8.77
Western blot
Coverage
(%)
MEK1-hi
(area)
MEK1-low
(area)
Semi-quantitative LFQ scores:
10.2-fold higher in MEK1-hi
B.
Total proteins identified
372
(21%)
1,154
(64%)
287
(15%)
MEK1-low
MEK1-hi
n=1,526
n=1,441
# of proteins identified:
| | Total | Exclusive (%) |
| --- | --- | --- |
| MEK1-low | 1,526 | 372 (21) |
| MEK1-hi | 1,441 | 287 (15) |

## Slide 8
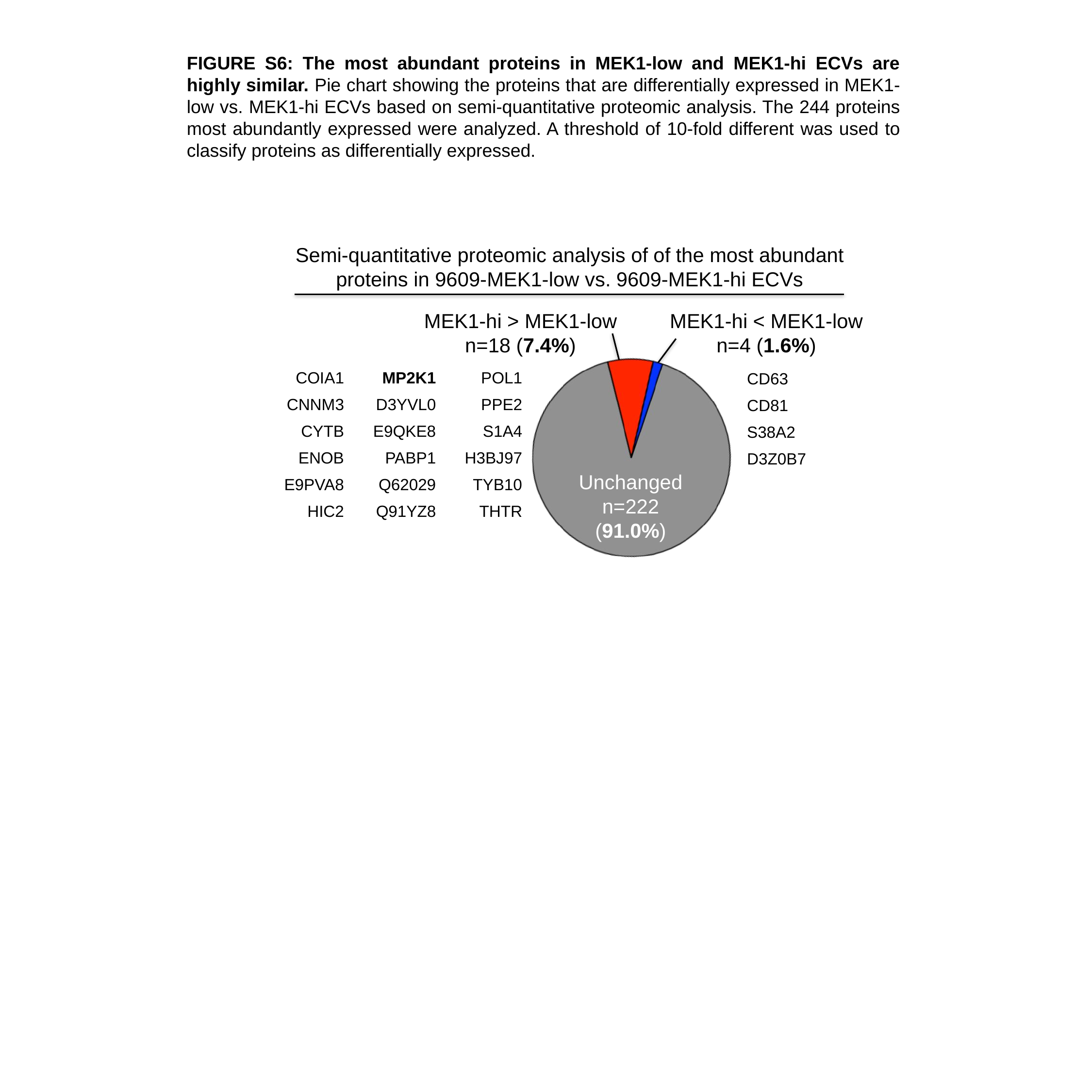

FIGURE S6: The most abundant proteins in MEK1-low and MEK1-hi ECVs are highly similar. Pie chart showing the proteins that are differentially expressed in MEK1-low vs. MEK1-hi ECVs based on semi-quantitative proteomic analysis. The 244 proteins most abundantly expressed were analyzed. A threshold of 10-fold different was used to classify proteins as differentially expressed.
Semi-quantitative proteomic analysis of of the most abundant proteins in 9609-MEK1-low vs. 9609-MEK1-hi ECVs
MEK1-hi < MEK1-low
n=4 (1.6%)
MEK1-hi > MEK1-low
n=18 (7.4%)
Unchanged
n=222 (91.0%)
| COIA1 | MP2K1 | POL1 |
| --- | --- | --- |
| CNNM3 | D3YVL0 | PPE2 |
| CYTB | E9QKE8 | S1A4 |
| ENOB | PABP1 | H3BJ97 |
| E9PVA8 | Q62029 | TYB10 |
| HIC2 | Q91YZ8 | THTR |
| CD63 |
| --- |
| CD81 |
| S38A2 |
| D3Z0B7 |

## Slide 9
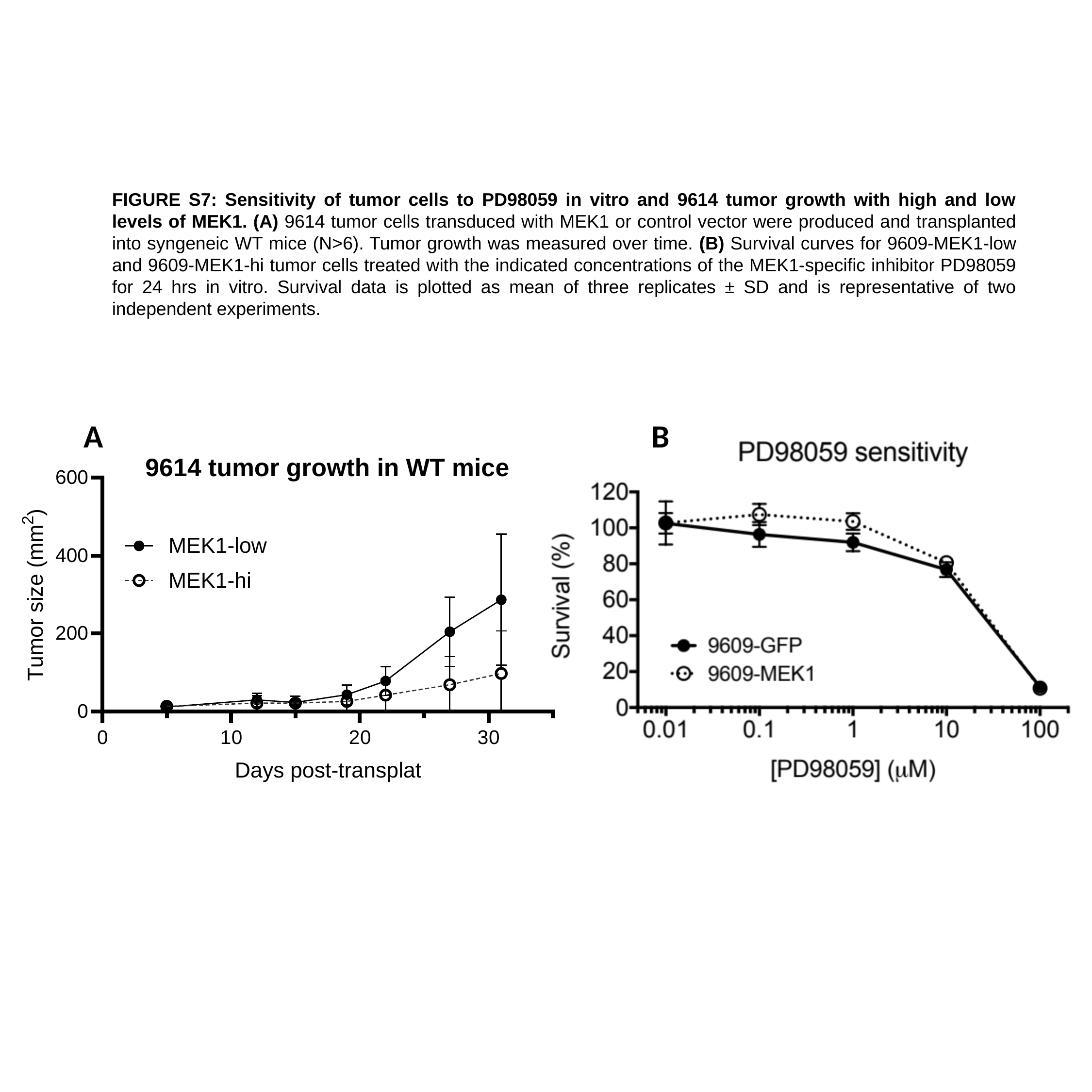

FIGURE S7: Sensitivity of tumor cells to PD98059 in vitro and 9614 tumor growth with high and low levels of MEK1. (A) 9614 tumor cells transduced with MEK1 or control vector were produced and transplanted into syngeneic WT mice (N>6). Tumor growth was measured over time. (B) Survival curves for 9609-MEK1-low and 9609-MEK1-hi tumor cells treated with the indicated concentrations of the MEK1-specific inhibitor PD98059 for 24 hrs in vitro. Survival data is plotted as mean of three replicates ± SD and is representative of two independent experiments.
A							 B

## Slide 10
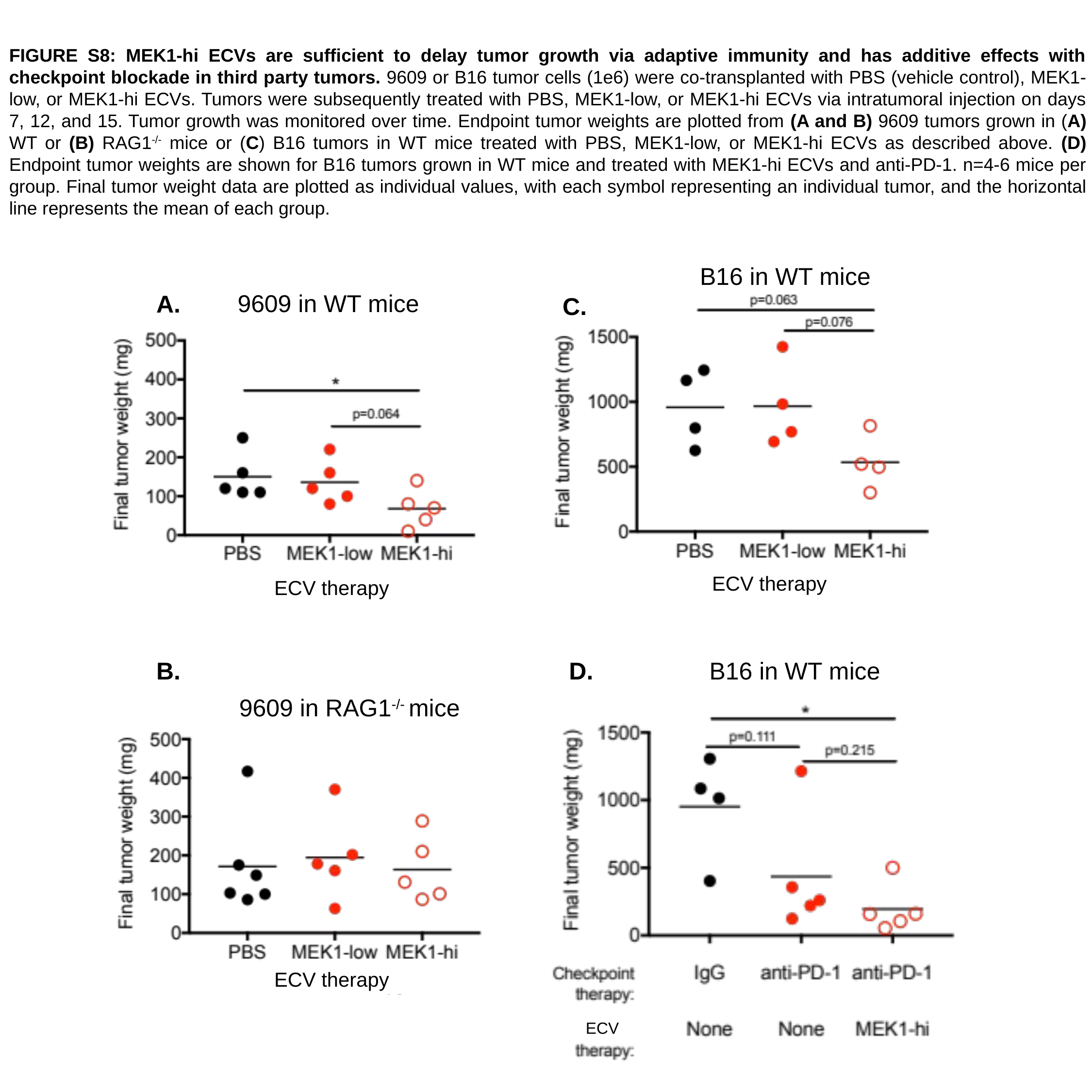

FIGURE S8: MEK1-hi ECVs are sufficient to delay tumor growth via adaptive immunity and has additive effects with checkpoint blockade in third party tumors. 9609 or B16 tumor cells (1e6) were co-transplanted with PBS (vehicle control), MEK1-low, or MEK1-hi ECVs. Tumors were subsequently treated with PBS, MEK1-low, or MEK1-hi ECVs via intratumoral injection on days 7, 12, and 15. Tumor growth was monitored over time. Endpoint tumor weights are plotted from (A and B) 9609 tumors grown in (A) WT or (B) RAG1-/- mice or (C) B16 tumors in WT mice treated with PBS, MEK1-low, or MEK1-hi ECVs as described above. (D) Endpoint tumor weights are shown for B16 tumors grown in WT mice and treated with MEK1-hi ECVs and anti-PD-1. n=4-6 mice per group. Final tumor weight data are plotted as individual values, with each symbol representing an individual tumor, and the horizontal line represents the mean of each group.
B16 in WT mice
9609 in WT mice
A.
C.
ECV therapy
ECV therapy
B16 in WT mice
B.
D.
9609 in RAG1-/- mice
ECV therapy
ECV

## Slide 11
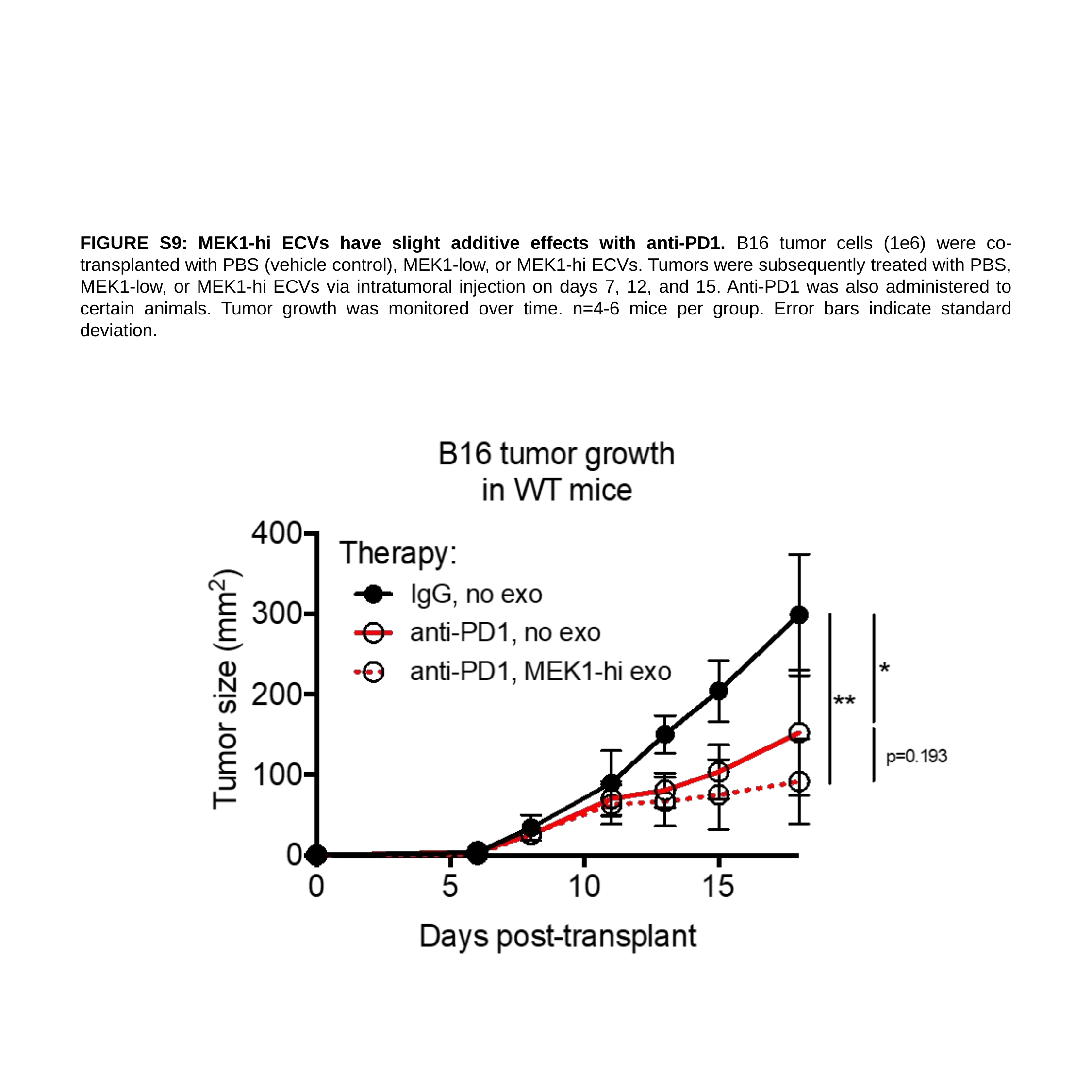

FIGURE S9: MEK1-hi ECVs have slight additive effects with anti-PD1. B16 tumor cells (1e6) were co-transplanted with PBS (vehicle control), MEK1-low, or MEK1-hi ECVs. Tumors were subsequently treated with PBS, MEK1-low, or MEK1-hi ECVs via intratumoral injection on days 7, 12, and 15. Anti-PD1 was also administered to certain animals. Tumor growth was monitored over time. n=4-6 mice per group. Error bars indicate standard deviation.

## Slide 12
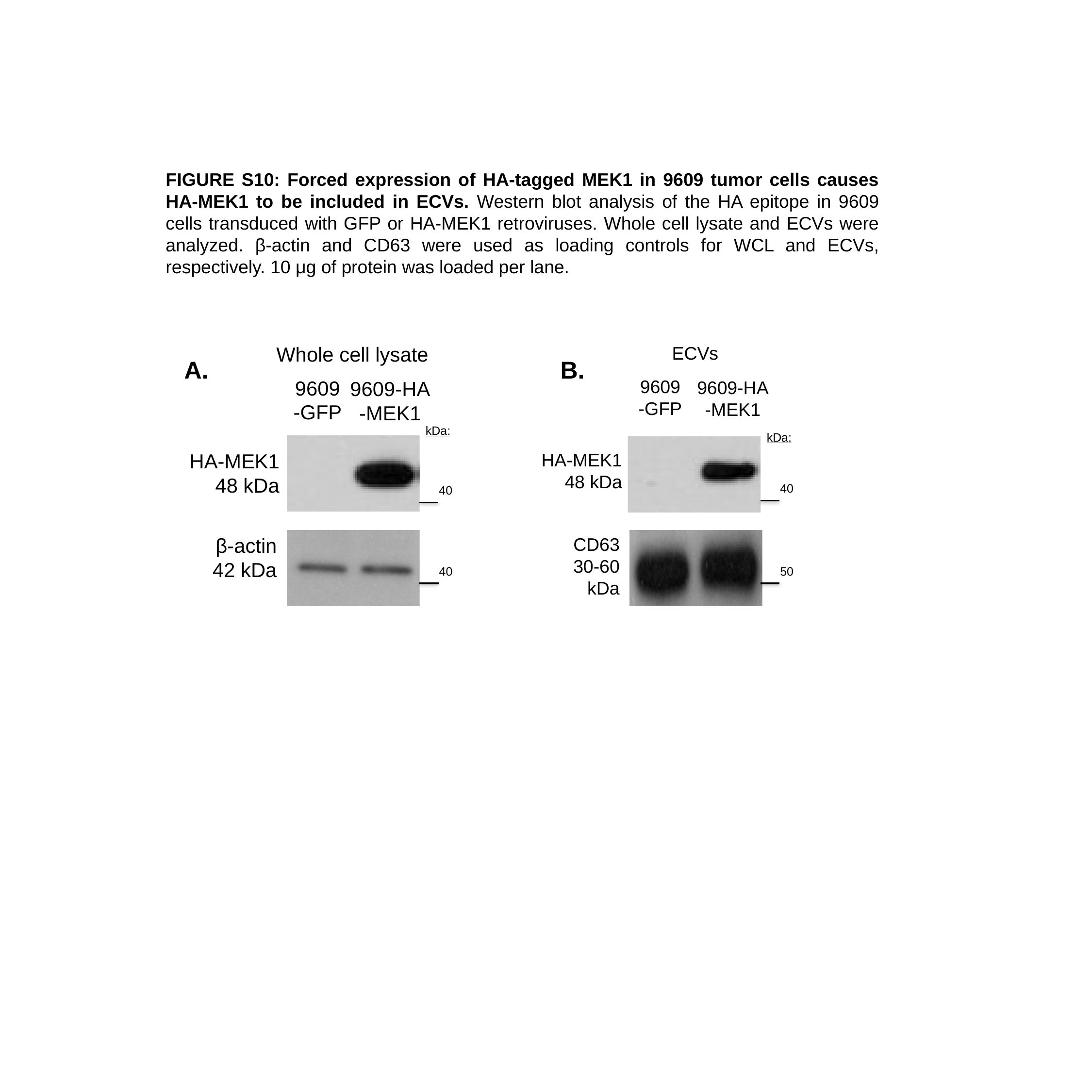

FIGURE S10: Forced expression of HA-tagged MEK1 in 9609 tumor cells causes HA-MEK1 to be included in ECVs. Western blot analysis of the HA epitope in 9609 cells transduced with GFP or HA-MEK1 retroviruses. Whole cell lysate and ECVs were analyzed. β-actin and CD63 were used as loading controls for WCL and ECVs, respectively. 10 μg of protein was loaded per lane.
Whole cell lysate
9609
-GFP
9609-HA
-MEK1
kDa:
HA-MEK1
48 kDa
40
β-actin
42 kDa
40
ECVs
9609
-GFP
9609-HA
-MEK1
kDa:
HA-MEK1
48 kDa
40
CD63
30-60 kDa
50
A.
B.

## Slide 13
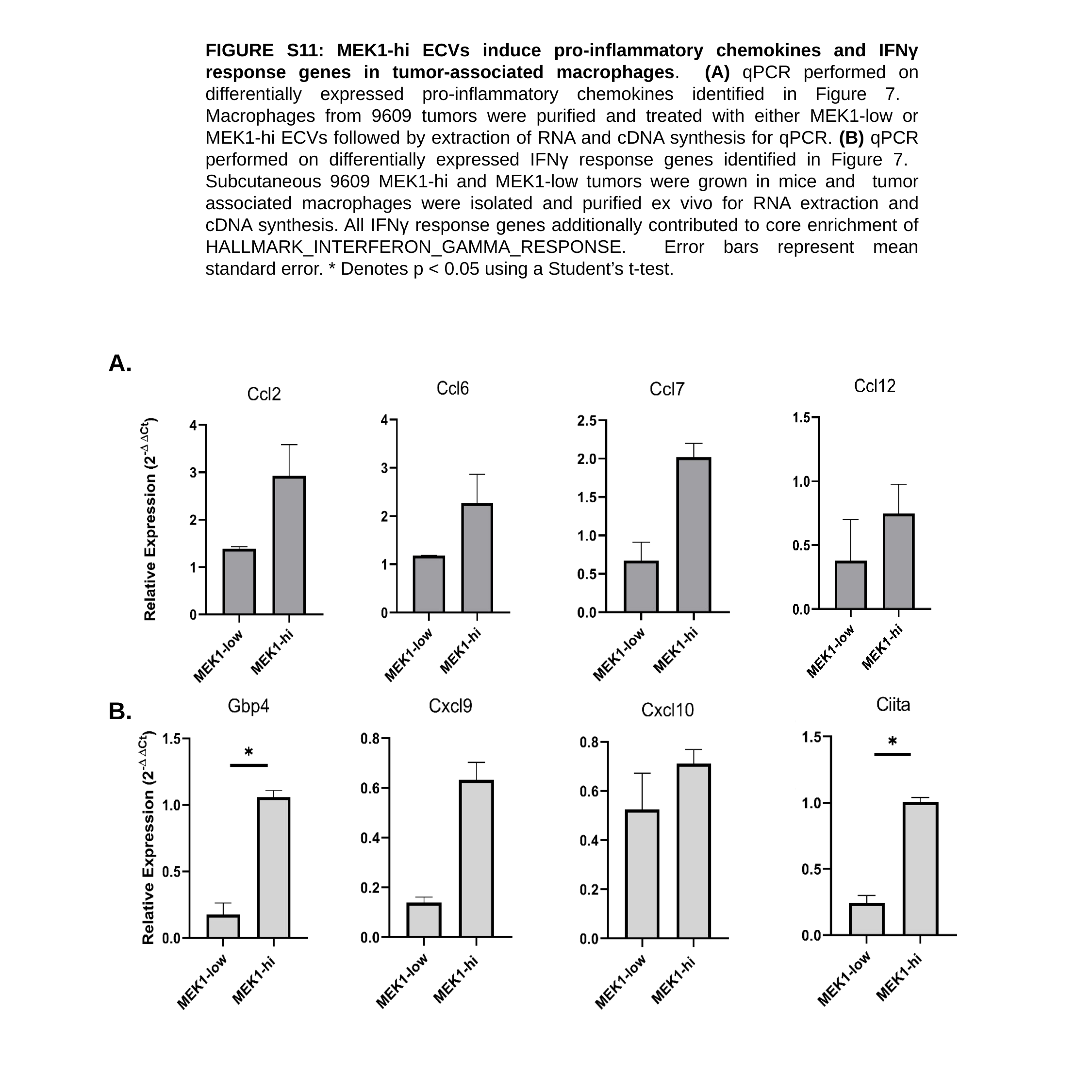

FIGURE S11: MEK1-hi ECVs induce pro-inflammatory chemokines and IFNγ response genes in tumor-associated macrophages. (A) qPCR performed on differentially expressed pro-inflammatory chemokines identified in Figure 7. Macrophages from 9609 tumors were purified and treated with either MEK1-low or MEK1-hi ECVs followed by extraction of RNA and cDNA synthesis for qPCR. (B) qPCR performed on differentially expressed IFNγ response genes identified in Figure 7. Subcutaneous 9609 MEK1-hi and MEK1-low tumors were grown in mice and tumor associated macrophages were isolated and purified ex vivo for RNA extraction and cDNA synthesis. All IFNγ response genes additionally contributed to core enrichment of HALLMARK_INTERFERON_GAMMA_RESPONSE. Error bars represent mean standard error. * Denotes p < 0.05 using a Student’s t-test.
A.
B.

## Slide 14
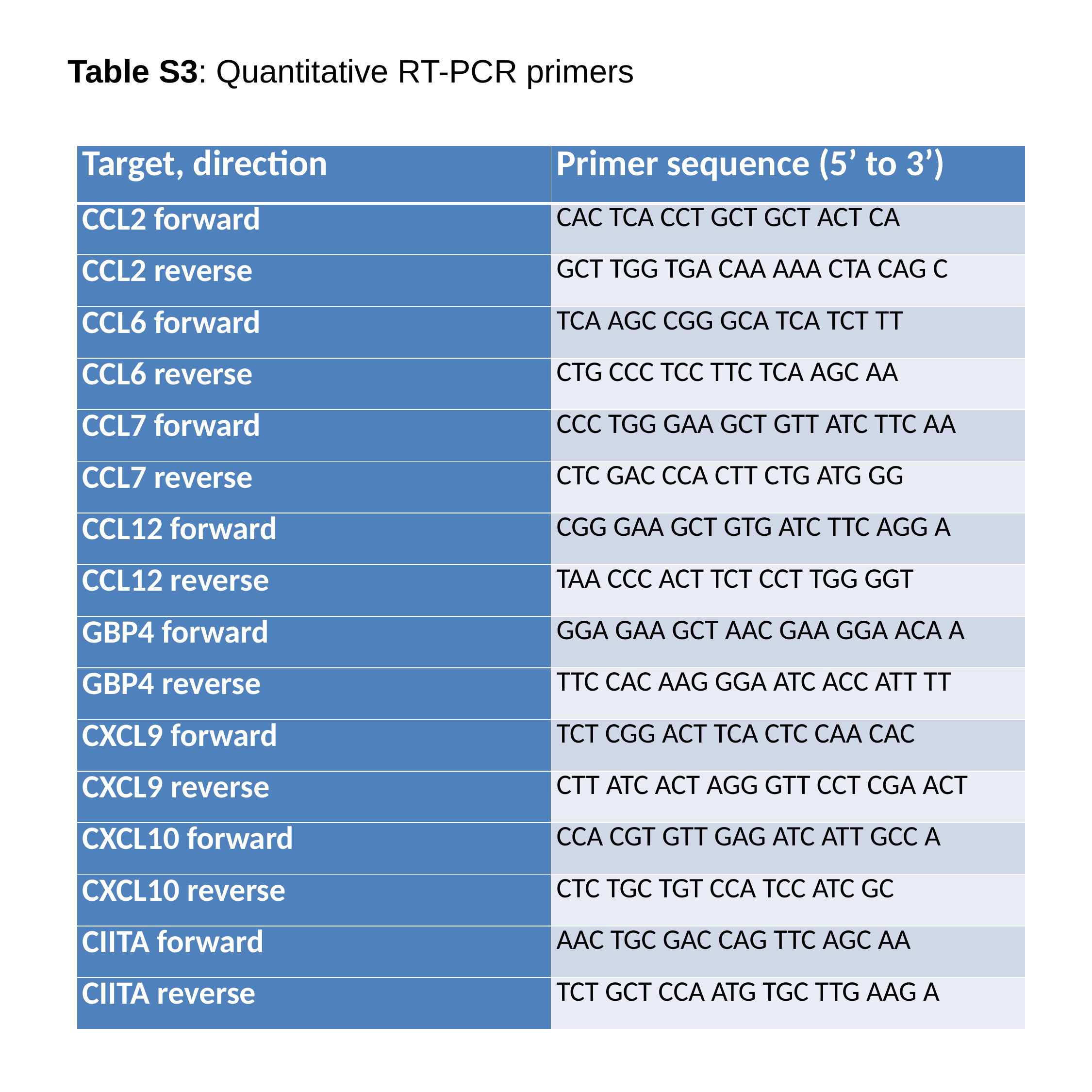

Table S3: Quantitative RT-PCR primers
| Target, direction | Primer sequence (5’ to 3’) |
| --- | --- |
| CCL2 forward | CAC TCA CCT GCT GCT ACT CA |
| CCL2 reverse | GCT TGG TGA CAA AAA CTA CAG C |
| CCL6 forward | TCA AGC CGG GCA TCA TCT TT |
| CCL6 reverse | CTG CCC TCC TTC TCA AGC AA |
| CCL7 forward | CCC TGG GAA GCT GTT ATC TTC AA |
| CCL7 reverse | CTC GAC CCA CTT CTG ATG GG |
| CCL12 forward | CGG GAA GCT GTG ATC TTC AGG A |
| CCL12 reverse | TAA CCC ACT TCT CCT TGG GGT |
| GBP4 forward | GGA GAA GCT AAC GAA GGA ACA A |
| GBP4 reverse | TTC CAC AAG GGA ATC ACC ATT TT |
| CXCL9 forward | TCT CGG ACT TCA CTC CAA CAC |
| CXCL9 reverse | CTT ATC ACT AGG GTT CCT CGA ACT |
| CXCL10 forward | CCA CGT GTT GAG ATC ATT GCC A |
| CXCL10 reverse | CTC TGC TGT CCA TCC ATC GC |
| CIITA forward | AAC TGC GAC CAG TTC AGC AA |
| CIITA reverse | TCT GCT CCA ATG TGC TTG AAG A |

## Slide 15
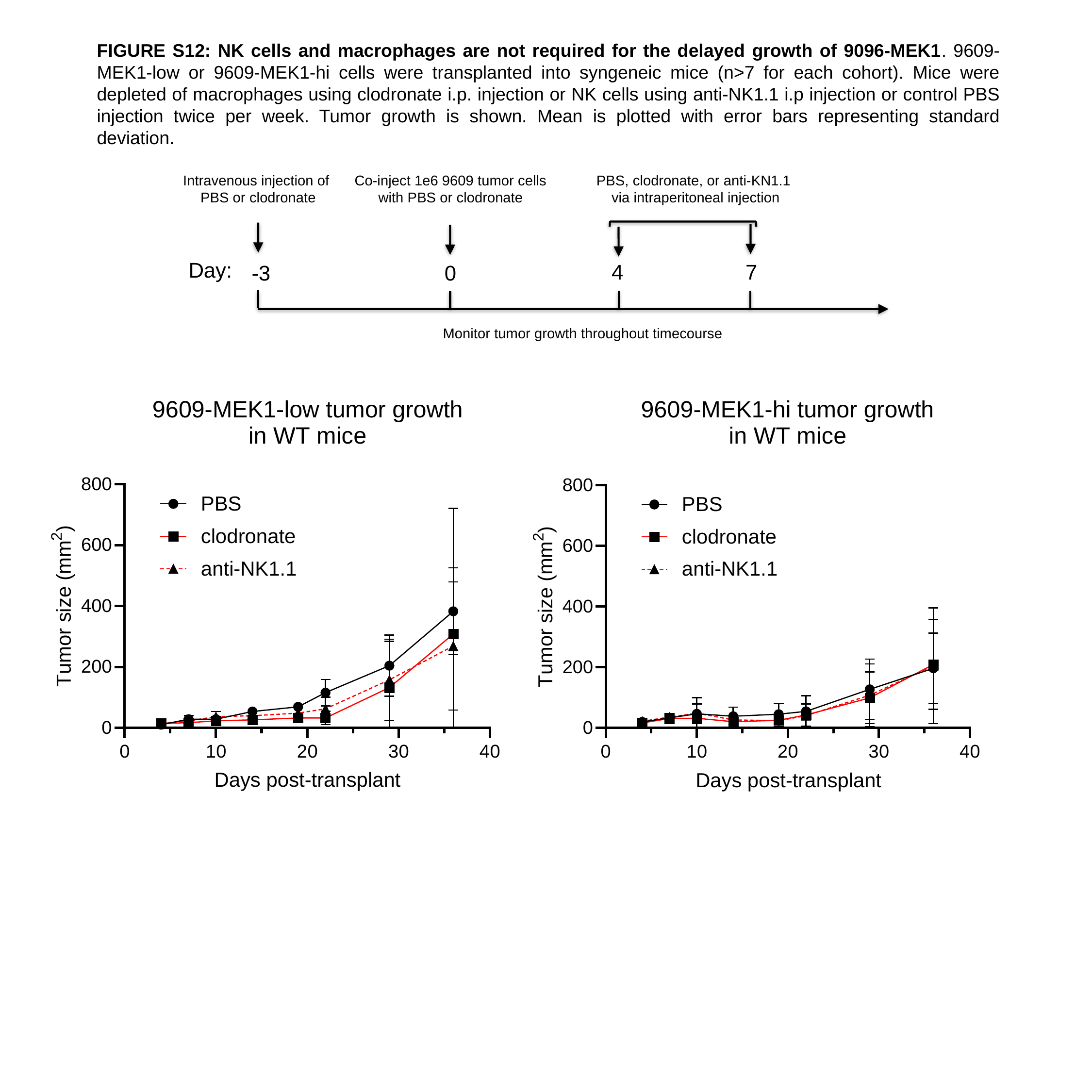

FIGURE S12: NK cells and macrophages are not required for the delayed growth of 9096-MEK1. 9609-MEK1-low or 9609-MEK1-hi cells were transplanted into syngeneic mice (n>7 for each cohort). Mice were depleted of macrophages using clodronate i.p. injection or NK cells using anti-NK1.1 i.p injection or control PBS injection twice per week. Tumor growth is shown. Mean is plotted with error bars representing standard deviation.
Intravenous injection of
PBS or clodronate
Co-inject 1e6 9609 tumor cells
with PBS or clodronate
PBS, clodronate, or anti-KN1.1
via intraperitoneal injection
4
7
-3
0
Monitor tumor growth throughout timecourse
Day:
